# Improved determination of the Higgs mass in the MSSM with heavy superpartners

**DOI:** 10.1140/epjc/s10052-017-4885-7

**Published:** 2017-05-20

**Authors:** Emanuele Bagnaschi, Javier Pardo Vega, Pietro Slavich

**Affiliations:** 1 Deutsches Elektronen-Synchrotron (DESY), 22607 Hamburg, Germany; 2 Abdus Salam International Centre for Theoretical Physics, Strada Costiera 11, 34151 Trieste, Italy; 3 SISSA International School for Advanced Studies and INFN Trieste, Via Bonomea 265, 34136 Trieste, Italy; 4 LPTHE, UPMC Univ. Paris 06, Sorbonne Universités, 4 Place Jussieu, 75252 Paris, France; 5 LPTHE, CNRS, 4 Place Jussieu, 75252 Paris, France

## Abstract

We present several advances in the effective field theory calculation of the Higgs mass in MSSM scenarios with heavy superparticles. In particular, we compute the dominant two-loop threshold corrections to the quartic Higgs coupling for generic values of the relevant SUSY-breaking parameters, including all contributions controlled by the strong gauge coupling and by the third-family Yukawa couplings. We also study the effects of a representative subset of dimension-six operators in the effective theory valid below the SUSY scale. Our results will allow for an improved determination of the Higgs mass and of the associated theoretical uncertainty.

## Introduction

At the price of doubling the particle content of the Standard Model (SM), supersymmetry (SUSY) provides elegant solutions to several open issues, including the stability of the electroweak (EW) scale, the nature of dark matter and the possibility of embedding the SM in a grand-unified gauge theory. Common features of supersymmetric extensions of the SM are an extended Higgs sector and the existence of tree-level relations between the quartic Higgs couplings and the other couplings of the considered model, which translate into predictions for the Higgs-boson masses. When radiative corrections are included, those predictions are sensitive to the whole particle spectrum of the model, and can be used to constrain its parameter space even before the discovery of SUSY particles.

In the minimal SUSY extension of the SM, or MSSM, the mass $m_h$ of the lightest Higgs scalar is bounded at tree level from above by $m_{\scriptscriptstyle Z}|\cos 2\beta |$, where $m_{\scriptscriptstyle Z}$ is the *Z*-boson mass and $\tan \beta \equiv v_2/v_1$ is the ratio of the vacuum expectation values (vevs) of the two Higgs doublets that participate in the breaking of the EW symmetry. However, as has been known [1–6] since the early 1990s, the tree-level upper bound on $m_h$ can be significantly raised by radiative corrections involving top quarks and their SUSY partners, the stop squarks. By now, the computation of radiative corrections to the MSSM Higgs masses^1^ is quite advanced: full one-loop corrections [7–13] and two-loop corrections in the limit of vanishing external momentum [14–27] are available, and the dominant momentum-dependent two-loop corrections [28–30] as well as the dominant three-loop corrections [31, 32] have also been obtained. Over the years, many of the known corrections have been implemented in widely used codes for the determination of the MSSM mass spectrum. In particular, FeynHiggs [33] includes full one-loop corrections to the Higgs masses from Ref. [13] and dominant two-loop corrections in the on-shell (OS) renormalization scheme from Refs. [17, 22–25, 29], whereas SoftSusy [34, 35], SuSpect [36] and SPheno [37, 38] include full one-loop corrections to the Higgs masses from Ref. [12] and dominant two-loop corrections in the ${ \overline{\mathrm{DR}}}$ scheme from Refs. [22–25, 39].

For the MSSM, both the discovery in 2012 [40, 41] of a SM-like Higgs boson with mass about 125 GeV [42] and the negative results of the searches for stop squarks at the LHC [43–48] favor scenarios with a SUSY mass scale $M_S$ in the TeV range. In particular, the observed value of the Higgs mass requires the radiative correction to the squared-mass parameter, $\Delta m_h^2\,$, to be at least as large as its tree-level value: if the stops are heavy enough, this can be realized via the dominant top/stop contributions, which are controlled by the top Yukawa coupling, $g_t \sim \mathcal{O}(1)$, and are enhanced by logarithms of the ratio between the stop and top masses. A further increase in $\Delta m_h^2$ can be obtained if the left–right stop mixing parameter $X_t$ is about twice the average stop mass. Roughly speaking, for $\tan \beta$ large enough to almost saturate the tree-level bound on the lightest-scalar mass, $m_h\approx 125$ GeV requires the average stop mass to be somewhere around 1 TeV for the “maximal” (i.e., most favorable) value of $X_t$, and above 10 TeV for vanishing $X_t$. However, when the SUSY scale is significantly larger than the EW scale, fixed-order calculations of $m_h$ such as the ones implemented in the codes mentioned above may become inadequate, because radiative corrections of order *n* in the loop expansion contain terms enhanced by as much as $\ln ^n(M_S/m_t)$–where we take the top mass as a proxy for the EW scale. Indeed, a possible symptom of such heavy-SUSY malaise is the fact that, in scenarios with TeV-scale stop masses and large stop mixing, the spread in the predictions of those codes for $m_h$ exceeds the theoretical accuracy of their (largely equivalent) two-loop calculations, which was estimated in the early 2000s to be about 3 GeV [49, 50] in what were then considered natural regions of the MSSM parameter space.

In the presence of a significant hierarchy between the SUSY scale and the EW scale, the computation of the Higgs mass needs to be reorganized in an effective field theory (EFT) approach: the heavy particles are integrated out at the scale $M_S$, where they only affect the matching conditions for the couplings of the EFT valid below $M_S$; the appropriate renormalization-group equations (RGEs) are then used to evolve those couplings between the SUSY scale and the EW scale, where the running couplings are related to physical observables such as the Higgs-boson mass and the masses of gauge bosons and fermions. In this approach, the computation is free of large logarithmic terms both at the SUSY scale and at the EW scale, while the effect of those terms is accounted for to all orders in the loop expansion by the evolution of the couplings between the two scales. More precisely, large corrections can be resummed to the (next-to)$^n$-leading-logarithmic (N$^n$LL) order by means of *n*-loop calculations at the SUSY and EW scales combined with $(n\!+\!1)$-loop RGEs. On the other hand, the common procedure of matching the MSSM to a renormalizable EFT – such as the plain SM – in the unbroken phase of the EW symmetry amounts to neglecting corrections suppressed by powers of $v^2/M_S^2\,$, where we denote by *v* the vev of a SM-like Higgs scalar. Those corrections can in fact be mapped to the effect of non-renormalizable, higher-dimensional operators in the EFT Lagrangian.

The EFT approach to the computation of the MSSM Higgs mass dates back to the early 1990s [51–53]. Over the years, it has also been exploited to determine analytically the coefficients of the logarithmic terms in $\Delta m_h^2$ at one [54], two [55–58] and even three [49, 59] loops, by solving perturbatively the appropriate systems of boundary conditions and RGEs. However, when the focus was on “natural” scenarios with SUSY masses of a few hundred GeV, the omission of $\mathcal{O}(v^2/M_S^2)$ terms limited the accuracy of the EFT approach, and the effect of the resummation of logarithmic corrections was not deemed important enough to justify abandoning the fixed-order calculations of the Higgs mass in favor of a complicated EFT set-up with higher-dimensional operators.^2^ More recently, an interest in “unnatural” scenarios such as split SUSY [61, 62] and high-scale SUSY (see, e.g., Ref. [63]), and then the LHC results pushing the expectations for the SUSY scale into the TeV range, have brought the EFT approach back into fashion. On the one hand, in Ref. [64] the authors of FeynHiggs combined the fixed-order calculation of $m_h$ implemented in their code with a resummation of the LL and NLL terms controlled exclusively by $g_t$ and by the strong gauge coupling $g_3$. On the other hand, three papers [65–67] presented updates of the traditional EFT calculation: the use of the state-of-the-art results collected in Ref. [68] for the SM part (i.e., three-loop RGEs and two-loop EW-scale matching conditions), together with the full one-loop and partial two-loop matching conditions at the SUSY scale, allow for a full NLL and partial NNLL resummation of the logarithmic corrections.^3^ Several public codes for the EFT calculation of the Higgs mass in the MSSM with heavy SUSY have also been released: SusyHD [69], based on Ref. [67]; MhEFT [70], based on Refs. [65, 71] and covering as well scenarios with a light two-Higgs-doublet model (THDM); HSSUSY [72, 73], a module of FlexibleSUSY [74] with the same essential features as the original SusyHD; FlexibleEFTHiggs [72, 73], which combines a full one-loop computation of $m_h$ with a LL resummation of the logarithmic corrections; finally, an EFT approach similar to the one of Ref. [73] was recently implemented in SPheno/SARAH [75].

In MSSM scenarios with stop masses of several TeV, where the effects of $\mathcal{O}(v^2/M_S^2)$ can be safely neglected, the theoretical uncertainty of the EFT prediction for the Higgs mass stems from missing terms of higher orders in the loop expansion, both in the calculation of the matching conditions at the SUSY scale and in the SM part of the calculation. In Refs. [66, 67] such uncertainty was estimated to be at most 1 GeV in a simplified MSSM scenario with degenerate SUSY masses of 10 TeV, $\tan \beta =20$ and vanishing $X_t$, where $m_h\approx 123.5$ GeV. In such scenario, the prediction for $m_h$ of the “hybrid” (i.e., fixed-order+partial NLL) calculation of Ref. [64] was about 3 GeV higher, well outside the theoretical uncertainty of the EFT result. In Refs. [67, 71] it was suggested that most of the discrepancy came from the determination of the coupling $g_t$ used in the resummation procedure, for which Ref. [64] omitted one-loop EW and two-loop QCD effects, consistently with the accuracy of the $m_h$ calculation in that paper. Those effects were later included in FeynHiggs, which now also allows for a full NLL and partial NNLL resummation of the logarithmic corrections [76]. In the simplified MSSM scenario mentioned above, the refinements in the resummation procedure of FeynHiggs reduce the discrepancy with the EFT prediction for $m_h$ to a few hundred MeV.

As mentioned earlier, MSSM scenarios with stop masses below a couple of TeV and large stop mixing – which are definitely more interesting from the point of view of LHC phenomenology – suffer from even larger spreads in the predictions of different codes for $m_h$. For example, in a benchmark point with degenerate SUSY masses of 1 TeV, $\tan \beta =20$, and $X_t$ varied so as to maximize $m_h$, the EFT calculation finds $m_h^{\mathrm{{\scriptscriptstyle max}}}\approx 123$ GeV, whereas SoftSusy, SuSpect and SPheno – which implement the same corrections to the Higgs masses, but differ in the determination of the running couplings – find $m_h^{\mathrm{{\scriptscriptstyle max}}}\approx 124.5-126.5$ GeV, and the latest version of FeynHiggs [77] finds $m_h^{\mathrm{{\scriptscriptstyle max}}}\approx 126-128$ GeV (depending on the code’s settings). However, in this case the comparison between the EFT prediction for $m_h$ and the various fixed-order (or hybrid) predictions is less straightforward than in scenarios with multi-TeV stop masses, because there is no obvious argument to favor one calculational approach over the others: the $\mathcal{O}(v^2/M_S^2)$ terms might or might not be negligible, and the logarithmic corrections might or might not be important enough to mandate their resummation. For all approaches, this unsatisfactory situation points to two urgent needs: first, to improve the calculation of $m_h$ with the inclusion of higher-order effects; second, to provide a better estimate of the theoretical uncertainty, tailored to the “difficult” region of the parameter space with stop masses about 1–2 TeV.

In this paper we take several steps towards an improved EFT determination of the Higgs mass in the MSSM with heavy superpartners. In particular, in Sect. 2 we compute the two-loop, $\mathcal{O}(g_t^6)$ contribution to the SUSY-scale matching condition for the quartic Higgs coupling – which was previously known only in simplified scenarios [21, 65, 67] – allowing for generic values of all the relevant SUSY-breaking parameters. We also include the two-loop contributions controlled by the bottom and tau Yukawa couplings, addressing some subtleties related to the presence of potentially large $\tan \beta$-enhanced corrections. Our new results bring the matching condition for the quartic Higgs coupling to the same level, in terms of an expansion in coupling constants, as the two-loop Higgs-mass calculations in SoftSusy, SuSpect and SPheno. In Sect. 3 we study instead the effects of a representative subset of dimension-six operators in the EFT. We obtain both an improvement in our prediction for $m_h$ in scenarios with stop masses about 1–2 TeV and a more-realistic estimate of the theoretical uncertainty associated to $\mathcal{O}(v^2/M_S^2)$ effects. The results presented in this paper have been implemented in modified versions of the codes SusyHD [69] and HSSUSY [72]. All the analytic formulas that proved too lengthy to be printed here are available upon request in electronic form.

## Two-loop matching of the quartic Higgs coupling

In this section we describe our calculation of the two-loop matching condition for the quartic Higgs coupling. We consider a set-up in which all SUSY particles as well as a linear combination of the two Higgs doublets of the MSSM are integrated out at a common renormalization scale $Q\approx M_S$, so that the EFT valid below the matching scale is just the SM. Using the conventions outlined in Sect. 2 of Ref. [66], the two-loop matching condition for the quartic coupling of the SM-like Higgs doublet *H* takes the form

1 $$\begin{aligned} \lambda (Q)= \frac{1}{4}\left[ g^2(Q)+ g^{\prime \,2}(Q)\right] \cos ^22\beta + \Delta \lambda ^{1\ell } + \Delta \lambda ^{2\ell }, \end{aligned}$$

where *g* and $g^\prime$ are the EW gauge couplings, $\beta$ can be interpreted as the angle that rotates the two original MSSM doublets into a light doublet *H* and a massive doublet *A*, and $\Delta \lambda ^{n\ell }$ is the *n*-loop threshold correction to the quartic coupling arising from integrating out the heavy particles at the scale $M_S$. The contributions to $\Delta \lambda ^{1\ell }$ controlled by the EW gauge couplings and by the top Yukawa coupling, for generic values of all SUSY parameters, were given in Ref. [66], completing and correcting earlier results of Refs. [78, 79]. For completeness, we report in the appendix the full result for the one-loop contributions of heavy scalars, including also terms controlled by the bottom and tau Yukawa couplings. However, the only one-loop contributions relevant to our computation of the two-loop threshold correction, where we will consider the “gaugeless” limit $g=g^\prime =0$, are those proportional to the fourth power of a third-family Yukawa coupling, which read

2 $$\begin{aligned} \Delta \lambda ^{g_f^4}= & {} \sum _{f=t,b,\tau }~ \frac{\hat{g}_f^4\, N^f_c}{(4\pi )^2}\,\nonumber \\&\times \left\{ \ln \frac{m_{\tilde{f}_{\scriptscriptstyle L}}^2m_{\tilde{f}_{\scriptscriptstyle R}}^2}{Q^4} + 2 \,\widetilde{X}_f \left[ \widetilde{F}_1 (x_f) -\frac{\widetilde{X}_f}{12} \,\widetilde{F}_2 (x_f)\right] \right\} ,\nonumber \\ \end{aligned}$$

where by $\hat{g}_f$ we denote SM-like Yukawa couplings,^4^ related to their MSSM counterparts $\hat{y}_f$ by $\hat{g}_t=\hat{y}_t\,\sin \beta \,$, $\hat{g}_b = \hat{y}_b \,\cos \beta$ and $\hat{g}_\tau = \hat{y}_\tau \,\cos \beta \,$. Moreover, for each fermion species *f*: $N^f_c$ is the number of colors; $(m_{\tilde{f}_{\scriptscriptstyle L}},m_{\tilde{f}_{\scriptscriptstyle R}})$ are the soft SUSY-breaking sfermion masses, i.e. $(m_{Q_3},m_{U_3})$, $(m_{Q_3},m_{D_3})$ and $(m_{L_3},m_{E_3})$ for stops, sbottoms and staus, respectively; $\widetilde{X}_f = X_f^2/(m_{\tilde{f}_{\scriptscriptstyle L}}m_{\tilde{f}_{\scriptscriptstyle R}})$, where $X_f = A_f-\mu \,r_f$, $A_f$ is the trilinear soft SUSY-breaking Higgs-sfermion coupling, $\mu$ is the Higgs-mass term in the MSSM superpotential, $r_t = \cot \beta$ and $r_b=r_{\tau }=\tan \beta \,$; $x_f = m_{\tilde{f}_{\scriptscriptstyle L}}/m_{\tilde{f}_{\scriptscriptstyle R}}\,$; finally, the loop functions $\widetilde{F}_1$ and $\widetilde{F}_2$ are defined in Appendix A of Ref. [66].

For what concerns the two-loop threshold correction $\Delta \lambda ^{2\ell }$, simplified results for the $\mathcal{O}(g_t^4\,g_3^2)$ and $\mathcal{O}(g_t^6)$ contributions, valid in the limit $m_{Q_3}=m_{U_3}=m_{\scriptscriptstyle A}=m_{\tilde{g}}\equiv M_S$ (where $m_{\scriptscriptstyle A}$ is the mass of the heavy Higgs doublet and $m_{\tilde{g}}$ is the gluino mass), were made available as far back as in Ref. [21]. Among the recent EFT analyses, Refs. [66, 67] obtained formulas for the $\mathcal{O}(g_t^4\,g_3^2)$ contributions valid for arbitrary values of all the relevant SUSY-breaking parameters. The $\mathcal{O}(g_t^6)$ contributions, on the other hand, were neglected in Ref. [66], while they were included in Refs. [65, 67] only through simplified formulas derived from those of Ref. [21]. In this paper we extend the calculations of Refs. [66, 67] to obtain all contributions to $\Delta \lambda ^{2\ell }$ controlled only by the third-family Yukawa couplings, again for arbitrary values of all the relevant SUSY-breaking parameters. Besides improving our knowledge of the $\mathcal{O}(g_t^6)$ contributions from two-loop diagrams involving stops, this allows us to properly account for sbottom and stau contributions that can become relevant at large values of $\tan \beta$. We also discuss how to obtain the $\mathcal{O}(g_b^4\,g_3^2)$ contributions from the known results for the $\mathcal{O}(g_t^4\,g_3^2)$ ones. Altogether, our results amount to a complete determination of $\Delta \lambda ^{2\ell }$ in the limit of vanishing EW gauge (and first-two-generation Yukawa) couplings.

### Outline of the calculation

The two-loop, Yukawa-induced threshold correction to the quartic Higgs coupling $\lambda$ at the matching scale *Q* can be expressed as

3 $$\begin{aligned} \Delta \lambda ^{2\ell } = \frac{1}{2}\,\left. {\frac{\partial ^4 \Delta V^{2\ell ,\,\mathrm{heavy}}}{\partial ^2H^\dagger \partial ^2 H}}\,\right| _{H=0} \!\!+~ \Delta \lambda ^{\mathrm{shift},\,f} + \Delta \lambda ^{\mathrm{shift},\tilde{f}},\nonumber \\ \end{aligned}$$

where by $\Delta V^{2\ell ,\,\mathrm{heavy}}$ we denote the contribution to the MSSM scalar potential from two-loop diagrams involving sfermions that interact with themselves, with Higgs doublets or with matter fermions and higgsinos only through the third-family Yukawa couplings, as well as from two-loop diagrams involving only the heavy Higgs doublet and matter fermions. The last two terms in Eq. (3) contain additional two-loop contributions that will be described below. In the following we will focus on the contributions to $\Delta \lambda ^{2\ell }$ that involve the top and bottom Yukawa couplings, and comment only briefly on the inclusion of the contributions that involve the tau Yukawa coupling, which are in general much smaller. However, we stress that the results that we implemented in SusyHD [69] and HSSUSY [72] (and that we make available upon request) do include the tau-Yukawa contributions through two loops.

In the gaugeless limit adopted in our calculation, the field-dependent mass spectrum of the particles that enter the relevant two-loop diagrams simplifies considerably: we can approximate the masses of the lightest Higgs scalar and of the would-be Goldstone bosons to zero, and the masses of all components (scalar, pseudoscalar and charged) of the heavy Higgs doublet to $m_{\scriptscriptstyle A}^2$; the charged and neutral components of the two higgsino doublets combine into Dirac spinors with degenerate mass eigenvalues $|\mu |^2$; the tree-level mixing angle in the CP-even sector is just $\alpha = \beta - \pi /2$. For the contributions to $\Delta V^{2\ell ,\,\mathrm{heavy}}$ that involve the top and bottom Yukawa couplings, we adapt the results used for the effective-potential calculation of the MSSM Higgs masses in Ref. [25].^5^ To compute the fourth derivative of the effective potential entering Eq. (3) we follow the approach outlined in section 2.3 of Ref. [66]: we express the stop and sbottom masses and mixing angles as functions of field-dependent top and bottom masses, $m_t = \hat{g}_t\, |H|$ and $m_b = \hat{g}_b \,|H|$, and obtain

4 $$\begin{aligned} \left. \frac{\partial ^4 \Delta V^{2\ell ,\,\mathrm{heavy}}}{\partial ^2H^\dagger \partial ^2 H}\,\right| _{H=0}= & {} \biggr [~ \hat{g}_t^4\,\left( 2 \,V_{tt}^{(2)} \,+\, 4\,m_t^2 \,V_{ttt}^{(3)} \,+\, m_t^4 \,V_{tttt}^{(4)}\right) \nonumber \\&+ \hat{g}_t^2\,\hat{g}_b^2 \left( 2 \,V_{tb}^{(2)} \,+\, 12\,m_t^2 \,V_{ttb}^{(3)} \,\right. \nonumber \\&\left. +\, 4\,m_t^4 \,V_{tttb}^{(4)}\,+\, 3\,m_t^2 \,m_b^2\, V_{ttbb}^{(4)} \right) \biggr ]_{m_t,m_b\,\rightarrow \,0}\nonumber \\&+ \biggr [\,t \,\longleftrightarrow \, b\biggr ], \end{aligned}$$

where the term in the last line is obtained from the terms in the first three lines by swapping top and bottom, and we used the shortcuts

5 $$\begin{aligned} V^{(k)}_{q_1\dots \, q_k} = \frac{\mathrm{d}^k \Delta V^{2\ell ,\,\mathrm{heavy}}}{\mathrm{d} m^2_{q_1}\dots \,\mathrm{d} m^2_{q_k}}. \end{aligned}$$

The derivatives of the field-dependent stop and sbottom parameters and the limit of vanishing top and bottom masses in Eq. (4) are obtained as described in Ref. [66]. As in the case of the $\mathcal{O}(g_t^4\,g_3^2)$ contributions, we find that the fourth derivative of the two-loop effective potential contains terms proportional to $\ln (m_q^2/Q^2)$, which would diverge for vanishing quark masses but cancel out against similar terms in the contribution denoted as $\Delta \lambda ^{\mathrm{shift},\,f}$ in Eq. (3). Indeed, above the matching scale the one-loop contribution to the quartic Higgs coupling from box diagrams with a top or bottom quark,

6 $$\begin{aligned} \delta \lambda ^{ g_q^4,\, q} = - \sum _{q=t,b} \frac{\hat{g}_q^4\,N_c}{(4\pi )^2}\, \left( 2\,\ln \frac{m_q^2}{Q^2} + 3 \right) , \end{aligned}$$

is expressed in terms of the MSSM couplings $\hat{g}_q$, whereas below the matching scale the same contribution is expressed in terms of the SM couplings $g_q$. To properly compute the two-loop, Yukawa-only part of the matching condition for the quartic Higgs coupling, we must re-express the MSSM couplings entering $\delta \lambda ^{ g_q^4,\, q}$ above the matching scale (including those implicit in $m_q$) according to $\hat{g}_q \,\rightarrow g_q\,(1+\Delta g_q^{\scriptscriptstyle Y})$, where $\Delta g_q^{\scriptscriptstyle Y}$ denotes the terms controlled by the Yukawa couplings in the threshold correction to $g_q$. In particular, we find for the top and bottom Yukawa couplings

7 $$\begin{aligned} \Delta g_t^{\scriptscriptstyle Y}= & {} - \frac{\hat{g}_t^2}{(4\pi )^2\sin ^2\beta } \,\left[ \, \frac{3}{4}\,\ln \frac{\mu ^2}{Q^2} \,+\,\frac{3}{8}\,\cos ^2\beta \, \left( 2\, \ln \frac{m_{\scriptscriptstyle A}^2}{Q^2} -1\right) \,\right. \nonumber \\&\left. +\,\widetilde{F}_6 \left( \frac{m_{Q_3}}{\mu }\right) \,+\, \frac{1}{2}\, \widetilde{F}_6 \left( \frac{m_{U_3}}{\mu } \right) \, \right] \nonumber \\&- \frac{\hat{g}_b^2}{(4\pi )^2\cos ^2\beta } \,\left[ \, \frac{1}{4}\,\ln \frac{\mu ^2}{Q^2} \,+\,\frac{1}{8}\,\sin ^2\beta \, \left( 2\, \ln \frac{m_{\scriptscriptstyle A}^2}{Q^2} -1\right) \,\right. \nonumber \\&\left. +\,\cos ^2\beta \, \left( \ln \frac{m_{\scriptscriptstyle A}^2}{Q^2} -1\right) \right. \nonumber \\&\left. \,+\, \frac{1}{2}\, \widetilde{F}_6 \left( \frac{m_{D_3}}{\mu } \right) \,\right. \nonumber \\&\left. +\, \frac{X_b\,\cot \beta }{2\,\mu }\,\widetilde{F}_9 \left( \frac{m_{Q_3}}{\mu },\frac{m_{D_3}}{\mu } \right) \right] - \frac{\delta Z_{\scriptscriptstyle H}^{\tilde{q}}}{2},\end{aligned}$$

8 $$\begin{aligned} \Delta g_b^{\scriptscriptstyle Y}= & {} - \frac{\hat{g}_b^2}{(4\pi )^2\cos ^2\beta } \,\left[ \, \frac{3}{4}\,\ln \frac{\mu ^2}{Q^2} \,+\,\frac{3}{8}\,\sin ^2\beta \, \left( 2\, \ln \frac{m_{\scriptscriptstyle A}^2}{Q^2} -1\right) \,\right. \nonumber \\&\left. +\,\widetilde{F}_6 \left( \frac{m_{Q_3}}{\mu }\right) \,+\, \frac{1}{2}\, \widetilde{F}_6 \left( \frac{m_{D_3}}{\mu } \right) \, \right] \nonumber \\&- \frac{\hat{g}_t^2}{(4\pi )^2\sin ^2\beta } \,\left[ \, \frac{1}{4}\,\ln \frac{\mu ^2}{Q^2} \,+\,\frac{1}{8}\,\cos ^2\beta \, \left( 2\, \ln \frac{m_{\scriptscriptstyle A}^2}{Q^2} -1\right) \,\right. \nonumber \\&\left. +\,\sin ^2\beta \, \left( \ln \frac{m_{\scriptscriptstyle A}^2}{Q^2} -1\right) \right. \nonumber \\&\left. \,+\, \frac{1}{2}\, \widetilde{F}_6 \left( \frac{m_{U_3}}{\mu } \right) \,\right. \nonumber \\&\left. +\, \frac{X_t\,\tan \beta }{2\,\mu }\,\widetilde{F}_9 \left( \frac{m_{Q_3}}{\mu },\frac{m_{U_3}}{\mu } \right) \right] - \frac{\delta Z_{\scriptscriptstyle H}^{\tilde{q}}}{2}, \end{aligned}$$

where the last term on the right-hand side of each equation reads, in a notation analogous to the one of Eq. (2),

9 $$\begin{aligned} \delta Z_{\scriptscriptstyle H}^{\tilde{q}} = -\sum _{q=t,b} \frac{\hat{g}_q^2\, N_c}{(4\pi )^2}~ \frac{\widetilde{X}_q}{6}~ \widetilde{F}_5 (x_q), \end{aligned}$$

and corresponds to the threshold correction to the light-Higgs WFR arising from squark loops. The loop functions $\widetilde{F}_5$, $\widetilde{F}_6$ and $\widetilde{F}_9$ are defined in appendix A of Ref. [66]. We also remark that Eqs. (7)–(9) assume that the angle $\beta$ entering the relations between the SM-like couplings $\hat{g}_q$ and their MSSM counterparts $\hat{y}_q$ is renormalized as described in Sect. 2.2 of Ref. [66], removing entirely the contributions of the off-diagonal WFR of the Higgs doublets. Combining the effects of the shifts in the Yukawa couplings with the renormalization of the Higgs fields (keeping into account also the field-dependent quark masses in the logarithms) we obtain the total contribution to $\Delta \lambda ^{2\ell }$ arising from the quark-box diagrams of Eq. (6),

10 $$\begin{aligned} \Delta \lambda ^{\mathrm{shift},\,f} = - \sum _{q=t,b} \frac{\hat{g}_q^4\,N_c}{(4\pi )^2} \left( 2\,\ln \frac{m_q^2}{Q^2} {+} 4 \right) \left( 4\,\Delta g_q^{\scriptscriptstyle Y} + 2\,\delta Z_{\scriptscriptstyle H}^{\tilde{q}}\right) , \end{aligned}$$

which cancels the logarithmic dependence on the quark masses of the derivatives of $\Delta V^{2\ell ,\,\mathrm{heavy}}$. We checked that the contributions in Eq. (4) that involve more than two derivatives of the two-loop effective potential cancel out completely against the shift of the corresponding contributions in the one-loop part – namely, the non-logarithmic term in the right-hand side of Eq. (6) – so that the final result for $\Delta \lambda ^{2\ell }$ can be related to the two-loop correction to the light-Higgs mass. This is the same “decoupling” property found in Ref. [66] for the $\mathcal{O}(g_t^4\,g_3^2)$ part of $\Delta \lambda ^{2\ell }$. Finally, it can be inferred from Eqs. (7)–(10) that the contribution of $\delta Z_{\scriptscriptstyle H}^{\tilde{q}}$ cancels out of $\Delta \lambda ^{\mathrm{shift},\,f}$.

The last term in Eq. (3), $\Delta \lambda ^{\mathrm{shift},\,\tilde{f}}$, arises from shifts in the sfermion contribution to the one-loop matching condition for the quartic Higgs coupling, Eq. (2). In particular, it contains terms arising from the WFR of the Higgs fields, which are not captured by the derivatives of $\Delta V^{2\ell ,\,\mathrm{heavy}}$, plus additional contributions that arise if we express the one-loop threshold correction in Eq. (2) in terms of the SM Yukawa couplings, $g_q$, instead of the MSSM ones, $\hat{g}_q$. We remark here that, while the shift of the Yukawa couplings in the quark-box diagrams of Eq. (6) is required for a consistent two-loop matching of the quartic Higgs coupling, an analogous shift in the squark contribution of Eq. (2) is to some extent a matter of choice. In Refs. [66, 67] the top Yukawa coupling entering the one-loop part of the threshold correction to the quartic Higgs coupling was interpreted as the SM one. Applying that choice to both the top and the bottom Yukawa couplings, we would find

11 $$\begin{aligned} \Delta \lambda ^{\mathrm{shift},\,\tilde{f}}= & {} \sum _{q=t,b}~ \frac{\hat{g}_q^4\, N_c}{(4\pi )^2}\,\left\{ \ln \frac{m_{\tilde{q}_{\scriptscriptstyle L}}^2m_{\tilde{q}_{\scriptscriptstyle R}}^2}{Q^4} \,\right. \nonumber \\&\left. +\,2 \, \widetilde{X}_q \left[ \widetilde{F}_1 (x_q) \,-\,\frac{\widetilde{X}_q}{12} \,\widetilde{F}_2 (x_q)\right] \right\} \nonumber \\&\Big (4\,\Delta g_q^{\scriptscriptstyle Y}+ 2\,\delta Z_{\scriptscriptstyle H}^{\tilde{q}}\Big ) , \end{aligned}$$

where again the contributions of the WFR of the Higgs fields cancel out against analogous terms in the shifts of the Yukawa couplings. After including in $\Delta \lambda ^{2\ell }$ the shifts in Eqs. (10) and (11), we checked that, in the limit of $g_b=0$ and $m_{Q_3}=m_{U_3}=m_{\scriptscriptstyle A}\equiv M_S$, the $\mathcal{O}(g_t^6)$ part of $\Delta \lambda ^{2\ell }$ coincides with the simplified result given in Eq. (21) of Ref. [67].

On the other hand, it is well known [80–82] that the relation between the bottom Yukawa coupling of the SM and its MSSM counterpart is subject to potentially large corrections enhanced by $\tan \beta$, which, in the gaugeless limit, arise from diagrams involving either gluino–sbottom or higgsino–stop loops. As discussed, e.g., in Ref. [83], these $\tan \beta$-enhanced terms can be “resummed” in the ${ \overline{\mathrm{DR}}}$-renormalized coupling of the MSSM by expressing it as

12 $$\begin{aligned} \hat{g}_b(Q) = \frac{ g_b(Q)}{1- \left( \Delta g_b^{s} + \Delta g_b^{\scriptscriptstyle Y}\right) }, \end{aligned}$$

where $g_b(Q)$ is the $\overline{\mathrm{MS}}$-renormalized coupling of the SM, extracted at the EW scale from the bottom mass and evolved up to the matching scale *Q* with SM RGEs, $\Delta g_b^{\scriptscriptstyle Y}$ is given in Eq. (7), and

13 $$\begin{aligned} \Delta g_b^{s}= & {} - \frac{g_3^2\,C_F}{(4\pi )^2}\,\left[ 1 \,+\,\ln \frac{m_{\tilde{g}}^2}{Q^2} \,+\, \widetilde{F}_6\left( \frac{m_{Q_3}}{m_{\tilde{g}}}\right) \,\right. \nonumber \\&\left. +\, \widetilde{F}_6\left( \frac{m_{D_3}}{m_{\tilde{g}}}\right) - \frac{X_b}{m_{\tilde{g}}}\, \widetilde{F}_9\left( \frac{m_{Q_3}}{m_{\tilde{g}}},\frac{m_{D_3}}{m_{\tilde{g}}}\right) \right] , \end{aligned}$$

where $C_F=4/3$ is a color factor, and we recall that $X_b=A_b-\mu \,\tan \beta$. In contrast with our treatment of the top Yukawa coupling, we will therefore choose to interpret the bottom Yukawa coupling entering the one-loop part of the threshold correction to the quartic Higgs coupling as the MSSM one, in order to absorb the $\tan \beta$-enhanced effects directly in $\Delta \lambda ^{1\ell }$. We recall that a similar approach was discussed in Refs. [18, 24, 25] in the context of the fixed-order calculation of the Higgs masses in the MSSM.

With our choice for the bottom Yukawa coupling entering $\Delta \lambda ^{1\ell }$, we must omit the term $4\,\Delta g_b^{\scriptscriptstyle Y}$ in the formula for $\Delta \lambda ^{\mathrm{shift},\,\tilde{f}}$, see Eq. (11), when computing the contributions to $\Delta \lambda ^{2\ell }$ controlled only by the top and bottom Yukawa couplings. Concerning the $\mathcal{O}(g_b^4\,g_3^2)$ contributions, they can be obtained from the $\mathcal{O}(g_t^4\,g_3^2)$ contributions computed in Refs. [66, 67] via

14 $$\begin{aligned} \Delta \lambda ^{g_b^4\,g_3^2}= & {} \Delta \lambda ^{g_t^4\,g_3^2} ~[\,t \,\rightarrow \, b\,]\nonumber \\&- 4\,\Delta g_b^s~\frac{\hat{g}_b^4\, N_c}{(4\pi )^2}\,\left\{ \ln \frac{m_{Q_3}^2m_{D_3}^2}{Q^4} \,+\, 2 \, \widetilde{X}_b \left[ \widetilde{F}_1 (x_b) \right. \right. \nonumber \\&\left. \left. -\,\frac{\widetilde{X}_b}{12} \,\widetilde{F}_2 (x_b)\right] \right\} , \end{aligned}$$

where the notation $[\,t \,\rightarrow \, b\,]$ in the first line represents the replacements $g_t \rightarrow g_b$, $X_t \rightarrow X_b$ and $m_{U_3} \rightarrow m_{D_3}$ in the formulas for the $\mathcal{O}(g_t^4\,g_3^2)$ contributions. We note that, in practice, our choice removes from $\Delta \lambda ^{2\ell }$ potentially large terms characterized by a higher power of $\tan \beta$ than of $\hat{g}_b$, i.e. terms scaling like $\hat{g}_b^4\,g_3^2\,\tan ^5\!\beta$ or like $\hat{g}_b^4\,\hat{g}_t^2\,\tan ^5\!\beta$.

We now comment on the inclusion of the contributions to $\Delta \lambda ^{2\ell }$ controlled by the tau Yukawa coupling. The two-loop contributions of $\mathcal{O}(g_\tau ^6)$, i.e. those involving only the tau Yukawa coupling, do not require a separate calculation, since they can be obtained from the top-only, $\mathcal{O}(g_t^6)$ ones via the replacements $g_t \rightarrow g_\tau$, $A_t\rightarrow A_{\tau }$, $N_c\rightarrow 1$, $m_{Q_3} \rightarrow m_{L_3}$, $m_{U_3} \rightarrow m_{E_3}$ and $\cos \beta \leftrightarrow \sin \beta$ (see also Ref. [25]). Indeed, as long as we neglect the EW gauge couplings, the threshold correction to the tau Yukawa coupling does not contain any $\tan \beta$-enhanced terms, and reads

15 $$\begin{aligned} \Delta g_\tau= & {} - \frac{\hat{g}_\tau ^2}{(4\pi )^2\cos ^2\beta } \left[ \frac{3}{4}\ln \frac{\mu ^2}{Q^2} {+}\frac{3}{8}\sin ^2\beta \left( 2\, \ln \frac{m_{\scriptscriptstyle A}^2}{Q^2} {-}1\right) \right. \nonumber \\&\left. +\,\widetilde{F}_6 \left( \frac{m_{L_3}}{\mu }\right) +\, \frac{1}{2}\, \widetilde{F}_6 \left( \frac{m_{E_3}}{\mu } \right) \right] -~ \frac{\delta Z_{\scriptscriptstyle H}^{\tilde{f}}}{2}, \end{aligned}$$

where the sfermion contribution to the Higgs WFR, $\delta Z_{\scriptscriptstyle H}^{\tilde{f}}$, is obtained including also the stau contribution (with $N_c=1$) in Eq. (9). We can therefore treat the tau Yukawa coupling in the same way as the top one, expressing the stau contribution to $\Delta \lambda ^{1\ell }$ in terms of the SM coupling $g_\tau$. In addition, “mixed” contributions to the two-loop effective potential controlled by both the tau and the bottom Yukawa couplings arise from diagrams that involve the quartic sbottom-stau coupling; see Appendix B of Ref. [50]. The corresponding contributions to $\Delta \lambda ^{2\ell }$ can be obtained directly from the derivatives of the effective potential (without additional shifts) with the procedure outlined around Eq. (4), after replacing $t \rightarrow \tau$ in the latter. Finally, the choice of using the MSSM coupling $\hat{g}_b$ in $\Delta \lambda ^{1\ell }$ spoils the cancellation of Higgs WFR effects in $\Delta \lambda ^{\mathrm{shift},\,\tilde{f}}$; see Eq. (11). As a result, when we take into account the $\mathcal{O}(g_\tau ^2)$ contribution from stau loops in $\delta Z_{\scriptscriptstyle H}^{\tilde{f}}$, we find an additional $\mathcal{O}(g_b^4\,g_\tau ^2)$ contribution to $\Delta \lambda ^{2\ell }$.

**Fig. 1:**
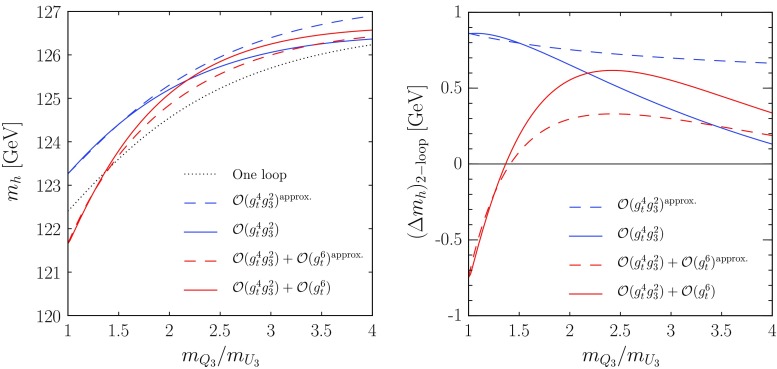
Effects of the top-Yukawa contributions to $\Delta \lambda ^{2\ell }$ in a scenario with non-degenerate SUSY masses, compared with approximate results obtained with degenerate masses. The *left plot* shows the predictions for $m_h$ as a function of the ratio of soft SUSY-breaking stop masses $m_{Q_3}/m_{U_3}\,$, while the *right plot* shows the shifts in $m_h$ induced by the two-loop contributions alone. The choices of MSSM parameters and the meaning of the *different curves* are described in the text

### Numerical examples

We now provide some illustration of the numerical impact of the newly computed two-loop corrections to the quartic Higgs coupling. To this purpose, we implemented those corrections in modified versions of the codes SusyHD [69] and HSSUSY [72]. All plots presented in this section were produced with HSSUSY, but we checked that fully analogous plots can be obtained with SusyHD. Small discrepancies in the predictions for $m_h$ arise from differences in the calculations implemented in the two codes, as discussed in Section 2.3 of Ref. [73], but they do not affect the qualitative behavior and relative importance of the new two-loop corrections. The SM input parameters used for HSSUSY in our studies, which we take from Ref. [84], are: the Fermi constant $G_F=1.16638\!\times \!10^{-5}$ GeV$^{-2}$; the Z boson mass $m_{\scriptscriptstyle Z}=91.1876$ GeV; the pole top mass $M_t^\mathrm{pole} = 173.21$ GeV; the $\overline{\mathrm{MS}}$-renormalized bottom mass $m_b(m_b)=4.18$ GeV; the tau mass $m_\tau = 1777$ MeV; finally, the strong and electromagnetic coupling constants in the five-flavor $\overline{\mathrm{MS}}$ scheme, $\alpha _s(m_{\scriptscriptstyle Z})=0.1181$ and $\alpha (m_{\scriptscriptstyle Z})=1/127.950$.

To start with, we omit all contributions to $\Delta \lambda ^{2\ell }$ controlled by the bottom and tau Yukawa couplings, and focus on the effect of extending the contributions controlled by the top Yukawa coupling to generic values of the relevant SUSY-breaking parameters. We consider a scenario in which all SUSY-particle masses are larger than one TeV, but the stop masses are not degenerate. In particular, we take $m_{U_3}= 1.5$ TeV and $m_{Q_3}= \kappa \,m_{U_3}$, where $\kappa$ is a scaling parameter that we vary in the range $1\le \kappa \le 4$. We also take $m_{\tilde{g}}=m_{\scriptscriptstyle A}= m_{U_3}$, $\,\mu = 4\,m_{U_3}$ and $\tan \beta =20$, and we fix $A_t$ via the “maximal” stop mixing condition $A_t-\mu \,\cot \beta = (6 \,m_{Q_3}\,m_{U_3})^{1/2}$. For the remaining MSSM parameters, which affect the one-loop part of the calculation, we set all sfermion masses other than those of the stops, as well as the EW gaugino masses, equal to $m_{U_3}$, and we take $A_b=A_{\tau }=A_t$. All of the MSSM parameters listed above – with the exception of $\tan \beta$, which is defined as described in Sect. 2.2 of Ref. [66] – are interpreted as ${ \overline{\mathrm{DR}}}$-renormalized parameters at the scale $Q=(m_{Q_3}\,m_{U_3})^{1/2}$.

In Fig. 1 we compare the predictions for $m_h$ obtained with the “exact” (i.e., valid for generic SUSY masses) formulas for the top-Yukawa contributions to $\Delta \lambda ^{2\ell }$ with “approximate” predictions obtained by replacing the scalar and gluino masses of our scenario with the degenerate masses $m_{Q_3}^\prime = m_{U_3}^\prime = m_{\scriptscriptstyle A}^\prime = m_{\tilde{g}}^\prime = (m_{Q_3}\,m_{U_3})^{1/2}$, and then using for the $\mathcal{O}(g_t^4\,g_3^2)$ and $\mathcal{O}(g_t^6)$ contributions to $\Delta \lambda ^{2\ell }$ the simplified formulas given in Refs. [66, 67], respectively. In particular, the dotted black line in the left plot of Fig. 1 represents the prediction for $m_h$, as a function of the stop mass ratio $\kappa = m_{Q_3}/m_{U_3}\,$, obtained by neglecting all two-loop contributions to the matching of the quartic Higgs coupling, and using the exact results from Refs. [66, 67] for the one-loop contributions; the dashed blue line includes also the simplified $\mathcal{O}(g_t^4\,g_3^2)$ contributions given in Eq. (36) of Ref. [66]; the solid blue line includes instead the exact $\mathcal{O}(g_t^4\,g_3^2)$ contributions from Refs. [66, 67]; the dashed red line includes, on top of the exact $\mathcal{O}(g_t^4\,g_3^2)$ contributions, the simplified $\mathcal{O}(g_t^6)$ contributions given in Eq. (21) of Ref. [67]; finally, the solid red line includes instead the exact $\mathcal{O}(g_t^6)$ contributions derived in this paper. In the right plot of Fig. 1 we show for clarity the effect on $m_h$ of the different implementations of the two-loop corrections alone, i.e. we show the difference between the (dashed or solid, blue or red) two-loop lines and the (dotted, black) one-loop line of the left plot. The meaning of each line in the right plot mirrors the one of the corresponding line in the left plot.

Figure 1 confirms that, as already noticed in Refs. [66, 67], the overall effect of the top-Yukawa contributions to $\Delta \lambda ^{2\ell }$ on the EFT predictions for $m_h$ in scenarios with multi-TeV stop masses is rather small, typically less than one GeV. However, the comparison between the dashed and solid lines in the plots of Fig. 1 shows that, in scenarios with non-degenerate mass spectra, the use of simplified formulas with an “average” SUSY mass can lead to a rather poor approximation of the exact results. In particular, the comparison between dashed and solid blue lines shows that by using Eq. (36) of Ref. [66] for the $\mathcal{O}(g_t^4\,g_3^2)$ corrections we would significantly overestimate their effect on $m_h$ when $\kappa \gtrsim 2$ in our scenario. In turn, the dashed and solid red lines show that, by using Eq. (21) of Ref. [67] for the $\mathcal{O}(g_t^6)$ corrections, we could entirely mischaracterize their effect on the Higgs mass: between the point where the solid blue line crosses the solid red one and the point where it crosses the dashed red one, the approximate calculation of the $\mathcal{O}(g_t^6)$ corrections gives a negative shift in $m_h$, while the exact calculation gives a positive shift. We remark, however, that the latter finding depends on the somewhat large value of $\mu$ adopted in our scenario: for smaller $\mu$ the quality of the approximation for the $\mathcal{O}(g_t^6)$ corrections would improve.

**Fig. 2:**
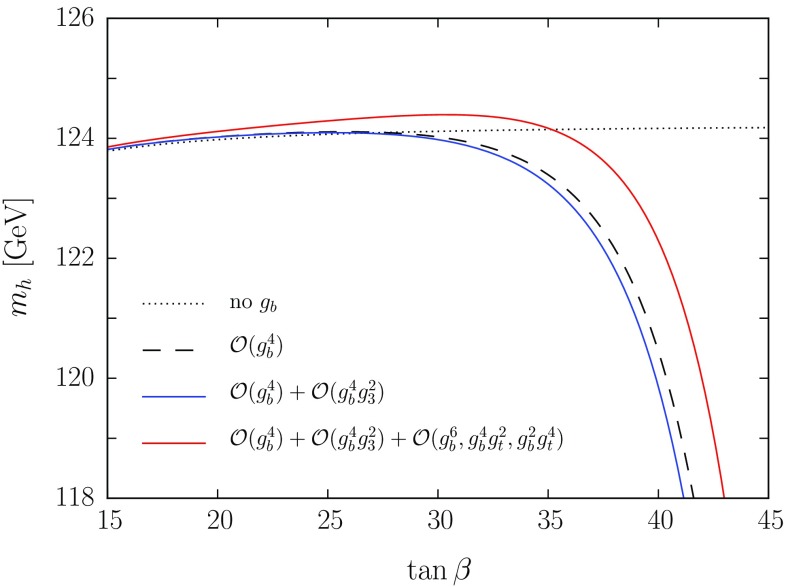
Predictions for $m_h$ as a function of $\tan \beta$ for different implementations of the corrections controlled by the *bottom* Yukawa coupling. We consider an MSSM scenario with all SUSY masses equal to $M_S=1.5~\mathrm{TeV}$ except $m_{\tilde{g}}= 2.5~\mathrm{TeV}$, and with $\mu =-1.5~\mathrm{TeV}$, $X_t= \sqrt{6}\,M_S$ and $A_b=A_{\tau }=A_t$. The meaning of the *different curves* is explained in the text

We now turn our attention to the effect of the threshold corrections to the quartic Higgs coupling controlled by the bottom Yukawa coupling. In Fig. 2 we show the EFT prediction for $m_h$ as a function of $\tan \beta$, in a simplified MSSM scenario with all soft SUSY-breaking masses of sfermions and EW gauginos, as well as the heavy Higgs-doublet mass $m_{\scriptscriptstyle A}$, set equal to $M_S= 1.5$ TeV, while the gluino mass is set to $m_{\tilde{g}}= 2.5$ TeV; the trilinear Higgs-stop coupling $A_t$ is fixed by the maximal mixing condition $A_t-\mu \,\cot \beta = \sqrt{6}\,M_S\,$, and $A_b=A_{\tau }=A_t$; finally, we take $\mu = -1.5$ TeV, to enhance the effect of the corrections controlled by the bottom Yukawa coupling. Indeed, negative values of the products $\mu \,m_{\tilde{g}}$ and $\mu A_t$ ensure that $\hat{g}_b$ – which we extract at the matching scale from the SM coupling $g_b$ via Eq. (12) – becomes larger for increasing $\tan \beta$, and possibly hits a pole as the denominator on the right-hand side of Eq. (12) approaches zero. Again, all soft SUSY-breaking parameters as well as $\mu$ are renormalized in the ${ \overline{\mathrm{DR}}}$ scheme at the matching scale $Q=M_S$.

The dotted black line in Fig. 2, which shows very little dependence on $\tan \beta$, represents the prediction for $m_h$ obtained by omitting the one- and two-loop corrections to the quartic Higgs coupling controlled by the bottom Yukawa coupling altogether; the dashed black line includes the one-loop $\mathcal{O}(g_b^4)$ contribution to $\Delta \lambda ^{1\ell }$, which, as discussed in Sect. 2.1, we express in terms of the MSSM coupling $\hat{g}_b\,$; the solid blue line includes also the two-loop $\mathcal{O}(g_b^4\,g_3^2)$ contributions to $\Delta \lambda ^{2\ell }$; finally, the solid red line includes also the two-loop $\mathcal{O}(g_b^6,\,g_b^4\,g_t^2,\,g_b^2\,g_t^4)$ contributions to $\Delta \lambda ^{2\ell }$. The comparison between the dashed black line and the solid blue and red lines shows that, when expressed in terms of the MSSM coupling $\hat{g}_b$, the $\mathcal{O}(g_b^4)$ contribution to $\Delta \lambda ^{1\ell }$ already determines the bulk of the dependence of $m_h$ on $\tan \beta$. Indeed, only at rather large $\tan \beta$, where the dependence becomes steep, can the $\mathcal{O}(g_b^4\,g_3^2)$ and $\mathcal{O}(g_b^6,\,g_b^4\,g_t^2,\,g_b^2\,g_t^4)$ contributions to $\Delta \lambda ^{2\ell }$ shift the prediction for $m_h$ by more than one GeV. Moreover, those corrections partially cancel out for our choice of MSSM parameters.

Finally, we recall that the strong dependence of $m_h$ on $\tan \beta$ depicted in Fig. 2 follows from our choice of signs for the products $\mu \,m_{\tilde{g}}$ and $\mu A_t\,$. If both of those products were positive instead of negative, the threshold correction $\left( \Delta g_b^{s} + \Delta g_b^{\scriptscriptstyle Y}\right)$ in Eq. (12) would suppress the MSSM coupling $\hat{g}_b$ – as well as the corresponding contributions to the quartic Higgs coupling and, in turn, to $m_h$ – at large values of $\tan \beta$. If the two products had opposite signs, the dependence of $m_h$ on $\tan \beta$ would hinge on whether it is $\Delta g_b^{s}$ or $\Delta g_b^{\scriptscriptstyle Y}$ that prevails in Eq. (12).

## On the effects of dimension-six operators

In MSSM scenarios with SUSY masses up to a couple of TeV, the effects suppressed by powers of $v^2/M_S^2$ – which are not accounted for when the EFT valid below the SUSY scale involves only renormalizable operators – might still be relevant. In the code SusyHD [67, 69] the uncertainty of the prediction for the Higgs mass associated to the omission of those effects is obtained by multiplying the contribution to $\Delta \lambda ^{1\ell }$ from each SUSY particle by a factor^6^ $(1\pm 2\, v^2/M_i^2)\,$, where $M_i$ is that particle’s mass. In a simplified scenario with $\tan \beta =20$, degenerate SUSY masses $M_i\equiv M_S$ and “maximal” $X_t = \sqrt{6}\,M_S$, the uncertainty arising from missing $\mathcal{O}(v^2/M_S^2)$ effects was thus estimated in Ref. [67] to be about 0.6 GeV for $M_S=1$ TeV, and to decrease rapidly for larger $M_S$. The total theoretical uncertainty of the EFT prediction for $m_h$, including also the effects of missing higher-order terms in the matching at the SUSY scale and in the SM part of the calculation, was estimated in Ref. [67] to be less than 2 GeV for $M_S=1$ TeV, where SusyHD finds $m_h\approx 123$ GeV. As mentioned in Sect. 1, in that scenario the predictions for the Higgs mass of various fixed-order (or hybrid) codes differ form each other by several GeV, and in general lie outside the estimated uncertainty of the EFT result. In this section we aim to improve the EFT calculation of the Higgs mass at moderate values of $M_S$ by including some of the most important $\mathcal{O}(v^2/M_S^2)$ effects, and to appraise the existing estimate of the uncertainty associated to the missing ones.

### Outline of the calculation

In the EFT framework, the effects of $\mathcal{O}(v^2/M_S^2)$ in the predictions for physical observables such as the Higgs mass arise from non-renormalizable, dimension-six effective operators. The most general dimension-six Lagrangian respecting the field content and symmetries of the SM contains a large number of operators; see Refs. [85–89] for recent reviews. In this section we focus on the two operators that induce one-loop corrections to $m_h^2$ proportional to $g_{t}^{2}\,m_t^{4}/M_S^2$ and two-loop corrections proportional to $g_{t}^{2}\,g_{3}^{2}\,m_t^4/M_S^2$, i.e. the terms suppressed by $m_t^{2}/M_S^2$  in what are usually denoted as one-loop $\mathcal{O}(\alpha _t)$ and two-loop $\mathcal{O}(\alpha _t\alpha _s)$ corrections to the Higgs mass, where $\alpha _{t} \equiv g_{t}^{2}/(4\pi )$ and $\alpha _{s} \equiv g_{3}^{2}/(4\pi )$. We write the Lagrangian of the SM extended by dimension-six operators as

16 $$\begin{aligned} \mathcal{L}_{\scriptscriptstyle \mathrm{EFT}} = \mathcal{L}_{\scriptscriptstyle \mathrm{SM}} - c_6\,|H|^6 + \left( \, c_t\,|H|^2 \,\overline{t_{\scriptscriptstyle R}} \, H^{\scriptscriptstyle T}\epsilon \,q_{\scriptscriptstyle L}+ \mathrm{h.c.}\, \right) , \end{aligned}$$

where $q_{\scriptscriptstyle L}$ and $t_{\scriptscriptstyle R}$ are third-generation quarks, $\epsilon$ is the antisymmetric tensor (with $\epsilon _{12}=1$) acting on the *SU*(2) indices, and, to fix our notation,

17 $$\begin{aligned} \mathcal{L}_{\scriptscriptstyle \mathrm{SM}} ~\supset ~ - m_{{\scriptscriptstyle H}}^2\,|H|^2 -\frac{\lambda }{2}\,|H|^4 + \left( \, g_t\,\overline{t_{\scriptscriptstyle R}} \, H^{\scriptscriptstyle T}\epsilon \, q_{\scriptscriptstyle L}~+~ \mathrm{h.c.}\, \right) . \end{aligned}$$

We stress that the choice of considering only the two dimension-six operators shown in Eq. (16) implies that our treatment of the $\mathcal{O}(v^2/M_S^2)$ effects is by no means complete, even when we restrict the calculation to the “gaugeless” limit $g\!=\!g^\prime \!=\!0$. Indeed, to account for the terms proportional to $g_t^4\,m_t^4/M_S^2$ , which are part of the two-loop $\mathcal{O}(\alpha _t^2)$ corrections to $m_h^2$ already included in most fixed-order codes, we should include in Eq. (16) also dimension-six operators that correct the kinetic term of the Higgs doublet.^7^ Concerning the resummation of the $\mathcal{O}(v^2/M_S^2)$ logarithmic corrections to $m_h^2$ beyond two loops, even to account only for the effects controlled by the highest powers of $g_3$ – i.e., the $(n\!+\!1)$-loop terms proportional to $g_t^2\,g_3^{2n}\,m_t^4/M_S^2\,\ln ^n(M_S/m_t)$ – we should include in Eq. (16) a set of dimension-six operators involving gluons.^8^ However, it must be kept in mind that the suppression by a factor $m_t^2/M_S^2$ implies that, for those corrections to be relevant, the argument of the resummed logarithms cannot be too large. As a result, there is no guarantee that the three-loop (and higher) logarithmic effects of $\mathcal{O}(v^2/M_S^2)$ that we could account for via resummation are more important than other effects that we are neglecting, such as, e.g., non-logarithmic three-loop corrections unsuppressed by $m_t^2/M_S^2$. The sure benefits of extending the SM Lagrangian with the two dimension-six operators of Eq. (16) are that *i*) we include in our calculation of the Higgs mass the $\mathcal{O}(v^2/M_S^2)$ part of one- and two-loop corrections that are known to be among the most significant ones, and *ii*) we can exploit our knowledge of the size of those corrections to estimate the theoretical uncertainty associated to other $\mathcal{O}(v^2/M_S^2)$ effects that we are neglecting.

The boundary conditions on the Wilson coefficients $c_6$ and $c_t$ are obtained by matching the EFT Lagrangian with the full MSSM Lagrangian at a renormalization scale $Q\approx M_S$. We start by remarking that those two coefficients receive contributions already at the tree level, controlled by the EW gauge couplings and generated when the heavy Higgs doublet – whose mass we denote by $m_{\scriptscriptstyle A}$ – is integrated out of the MSSM Lagrangian:

18 $$\begin{aligned} c_6^\mathrm{tree}= & {} -\frac{(g^2+g^{\prime \,2})^2}{64\,m_{\scriptscriptstyle A}^2}\,\sin ^24\beta ,\nonumber \\ c_t^\mathrm{tree}= & {} \frac{g_t\,(g^2+g^{\prime \,2})}{8\,m_{\scriptscriptstyle A}^2}\,\sin 4\beta \,\cot \beta . \end{aligned}$$

However, in the limit of large $\tan \beta$ both contributions scale like $1/\tan ^2\beta$. For $\tan \beta \gtrsim 10\,$, which we require to saturate the tree-level prediction for $m_h$ and allow for stop masses around one TeV, the resulting suppression makes the tree-level contributions to $c_6$ and $c_t$ numerically comparable with the one-loop contributions controlled by the EW gauge couplings, which we are not considering in our study. We will therefore omit the tree-level contributions of Eq. (18) altogether in what follows, and we now move on to summarizing our calculation of the one- and two-loop matching conditions relevant to the $\mathcal{O}(\alpha _t)$ and $\mathcal{O}(\alpha _t\alpha _s)$ corrections to the Higgs mass.

**Matching of** $\mathbf {c_t}$: The one-loop matching condition for $c_t$ can be derived by equating the expressions for the pole top-quark mass computed below and above the matching scale:

19 $$\begin{aligned} M_t^{\scriptscriptstyle \mathrm{pole}}= & {} g_t\,v \,+\, c_t^{1\ell }\,v^3 \,-\, \Sigma _t^{1\ell }(m_t)^{\scriptscriptstyle \mathrm{EFT,\overline{\mathrm{MS}}}}\nonumber \\= & {} \hat{g}_t\,\hat{v} \,-\, \Sigma _t^{1\ell }(m_t)^{\scriptscriptstyle \mathrm{MSSM,{ \overline{\mathrm{DR}}}}}, \end{aligned}$$

where $\Sigma _t^{1\ell }(m_t)$ is the one-loop self energy of the top quark computed with the external momentum $p^2=m_t^2$, and *v* is the Higgs vev in the EFT, while $\hat{v} = \sqrt{v_1^2 + v_2^2}$ is the corresponding quantity in the MSSM. We adopt as usual the ${ \overline{\mathrm{DR}}}$ scheme for the MSSM calculation and the $\overline{\mathrm{MS}}$ scheme for the EFT calculation (note, however, that $c_t^{1\ell }$ is the same in both schemes). We focus here on the $\mathcal{O}(g_3^2)$ and $\mathcal{O}(g_t^3\,g_3^2)$ contributions to the matching conditions for $g_t$ and $c_t$, respectively, which are necessary to reproduce the two-loop $\mathcal{O}(\alpha _t\alpha _s)$ corrections to the Higgs mass. Defining $\hat{g}_t(Q) = g_t(Q) \,(1+\Delta g_t^s)$, and considering that the distinction between *v* and $\hat{v}$ does not matter at $\mathcal{O}(g_3^2)$, we can extract $\Delta g_t^s$ and $c_t^{1\ell }$ from the terms of $\mathcal{O}(v)$ and $\mathcal{O}(v^3)$, respectively, in an expansion of the stop–gluino contribution to the top self energy in powers of *v*. Starting from Eq. (B2) of Ref. [22] for the unexpanded self energy, we find

20 $$\begin{aligned} \Delta g_t^{s}= & {} - \frac{g_3^2}{(4\pi )^2}\,C_F\,\left[ 1 \,+\,\ln \frac{m_{\tilde{g}}^2}{Q^2} \,\right. \nonumber \\&+\,\left. \, \widetilde{F}_6(x_{\scriptscriptstyle Q}) \,+\, \widetilde{F}_6(x_{\scriptscriptstyle U}) \,-\, \frac{X_t}{m_{\tilde{g}}}\,\widetilde{F}_9(x_{\scriptscriptstyle Q},x_{\scriptscriptstyle U})\right] ,\end{aligned}$$

21 $$\begin{aligned} c_t^{1\ell }(Q)= & {} \frac{\hat{g}_t^3\,g_3^2}{(4\pi )^2}\, \frac{C_F}{m_{\tilde{g}}^2} \left\{ \, \frac{11 + x_{\scriptscriptstyle Q}^2\,(2\,x_{\scriptscriptstyle Q}^2-7)}{6\,(x_{\scriptscriptstyle Q}^2-1)^3} - \frac{2\,\ln x_{\scriptscriptstyle Q}}{(x_{\scriptscriptstyle Q}^2-1)^4}\right. \nonumber \\&+\,\left( \frac{X_t}{m_{\tilde{g}}}-\frac{X_t^2}{2\,m_{\tilde{g}}^2}\right) \left[ \frac{x_{\scriptscriptstyle Q}^2-5}{2\,(x_{\scriptscriptstyle Q}^2-1)^2\,(x_{\scriptscriptstyle U}^2-1)}\right. \nonumber \\&\left. -\,\frac{4\,\ln x_{\scriptscriptstyle Q}}{(x_{\scriptscriptstyle Q}^2-1)^3\,(x_{\scriptscriptstyle Q}^2-x_{\scriptscriptstyle U}^2)}\right] \nonumber \\&\left. -\,\frac{2\,X_t^3}{m_{\tilde{g}}^3} \left[ \frac{1}{(x_{\scriptscriptstyle Q}^2-1)\,(x_{\scriptscriptstyle Q}^2-x_{\scriptscriptstyle U}^2)^2}\right. \right. \nonumber \\&\left. \left. -\, \frac{2\,(2\,x_{\scriptscriptstyle Q}^4-x_{\scriptscriptstyle Q}^2-x_{\scriptscriptstyle U}^2)\,\ln x_{\scriptscriptstyle Q}}{(x_{\scriptscriptstyle Q}^2-1)^2\,(x_{\scriptscriptstyle Q}^2-x_{\scriptscriptstyle U}^2)^3} \,\right] \,\right\} \nonumber \\&+\,\biggr [\,x_{\scriptscriptstyle Q}~\longleftrightarrow ~x_{\scriptscriptstyle U}\,\biggr ], \end{aligned}$$

where the functions $\widetilde{F}_6$ and $\widetilde{F}_9$ can be found in appendix A of Ref. [66], we defined $x_{\scriptscriptstyle Q}= m_{Q_3}/m_{\tilde{g}}$ and $x_{\scriptscriptstyle U}= m_{U_3}/m_{\tilde{g}}$ , and the term in the last line of Eq. (21) is obtained from the terms in the first five lines by swapping $x_{\scriptscriptstyle Q}$ and $x_{\scriptscriptstyle U}$. We note that the right-hand side of Eq. (21) does not depend explicitly on the scale *Q*. For the simplified choice $m_{Q_3}=m_{U_3}=m_{\tilde{g}}=M_S$, the $\mathcal{O}(g_t^3\,g_3^2)$ contribution to the matching condition for $c_t$ reduces to

22 $$\begin{aligned} c_t^{1\ell }(Q) = \frac{\hat{g}_t^3\,g_3^2}{(4\pi )^2}\, \frac{C_F}{12\,M_S^2} \left( 6 ~+~ 6\,\frac{X_t}{M_S} - 3\,\frac{X_t^2}{M_S^2} - 2\,\frac{X_t^3}{M_S^3}\right) ~. \end{aligned}$$

**Matching of** $\mathbf {c_6}$: The matching condition for the Wilson coefficient of the operator $|H|^6$ in Eq. (16) can, in analogy with the calculation of the matching condition for the quartic Higgs coupling described in Sect. 2.1, be obtained from the derivatives with respect to the Higgs field of the sfermion contributions to the effective potential of the MSSM. In particular, the $\mathcal{O}(g_t^6)$ contribution to the one-loop coefficient $c_6^{1\ell }$ at the matching scale *Q* reads

23 $$\begin{aligned} c_6^{1\ell }(Q) = \frac{1}{36}\, \left. \frac{\partial ^6 \Delta V^{1\ell ,\,\tilde{t}}}{\partial ^3H^\dagger \partial ^3 H}\,\right| _{H=0}, \end{aligned}$$

where $\Delta V^{1\ell ,\,\tilde{t}}$ is the stop contribution to the Coleman–Weinberg potential of the MSSM,

24 $$\begin{aligned} \Delta V^{1\ell ,\,\tilde{t}}=\frac{N_c}{(4\pi )^2}\,\sum _{i=1,2}\, \frac{m_{\tilde{t}_i}^4}{2}\,\left( \ln \frac{m_{\tilde{t}_i}^2}{Q^2} - \frac{3}{2}\right) . \end{aligned}$$

As outlined in Section 2.3 of Ref. [66], the derivatives of $\Delta V^{1\ell ,\,\tilde{t}}$ with respect to the Higgs field can easily be computed after expressing the stop masses $m_{\tilde{t}_i}^2$ as functions of the field-dependent top mass $m_t= \hat{g}_t\,|H|$, leading to

25 $$\begin{aligned} \left. \frac{\partial ^6 \Delta V^{1\ell ,\,\tilde{t}}}{\partial ^3H^\dagger \partial ^3 H}\,\right| _{H=0}= & {} ~ \hat{g}_t^6\,\Big [6\,V^{(3)}_{ttt}\,+\,18\,m_t^2\,V^{(4)}_{tttt}\,\nonumber \\&+\, 9\,m_t^4\,V^{(5)}_{ttttt}\,+\,m_t^6\,V^{(6)}_{tttttt} \Big ]_{m_t\rightarrow 0}, \end{aligned}$$

where we used for the derivatives of the one-loop potential shortcuts analogous to those defined in Eq. (5) for the derivatives of the two-loop potential. Explicitly, we find

26 $$\begin{aligned} c_6^{1\ell }(Q)= & {} \frac{\hat{g}_t^6}{(4\pi )^2}\,\frac{N_c}{m_{Q_3}m_{U_3}}\, \left\{ \frac{1+x_t^2}{6\,x_t}-\frac{\widetilde{X}_t}{2}\right. \nonumber \\&+\,\left. \widetilde{X}_t^2\,\left[ \,\frac{x_t\,(1+x_t^2)}{2\,(1-x_t^2)^2} \,+\,\frac{2\,x_t^3\,\ln x_t}{(1-x_t^2)^3}\,\right] \right. \nonumber \\&-\,\widetilde{X}_t^3\,\left[ \,\frac{x_t^2\,(1+10\,x_t^2+x_t^4)}{6\,(1-x_t^2)^4} \,\right. \nonumber \\&\left. \left. +\,\frac{2\,x_t^4\,(1+x_t^2)\,\ln x_t}{(1-x_t^2)^5}\,\right] \right\} , \end{aligned}$$

where, following the notation of Eq. (2), we defined $x_t = m_{Q_3}/m_{U_3}$ and $\widetilde{X}_t = X_t^2/ (m_{Q_3}m_{U_3})$. Eq. (26) agrees with the corresponding results in Refs. [91, 92],^9^ which employed the method known as “covariant derivative expansion” [93–95] to compute the one-loop matching conditions for all bosonic dimension-six operators induced by integrating the squarks out of the MSSM Lagrangian. In the limit of degenerate squark masses $m_{Q_3}=m_{U_3}=M_S$, the $\mathcal{O}(g_t^6)$ contribution to $c_6^{1\ell }$ reduces to

27 $$\begin{aligned} c_6^{1\ell }(Q) = \frac{\hat{g}_t^6}{(4\pi )^2}\,\frac{N_c}{M_S^2}\, \left( \frac{1}{3} \,-\,\frac{X_t^2}{2\,M_S^2} \,+\, \frac{X_t^4}{6\,M_S^4} \,-\,\frac{X_t^6}{60\,M_S^6}\,\right) , \end{aligned}$$

in agreement with the result presented long ago in Ref. [53].

The $\mathcal{O}(g_t^6g_3^2)$ contribution to the two-loop coefficient $c_6^{2\ell }$ at the matching scale *Q* reads

28 $$\begin{aligned} c_6^{2\ell }(Q) = \frac{1}{36}\, \left. \frac{\partial ^6 \Delta V^{2\ell ,\,\tilde{t}}}{\partial ^3H^\dagger \partial ^3 H}\,\right| _{H=0} -~\delta c_6^{\scriptscriptstyle \mathrm{EFT}} ~+~\delta c_6^{\mathrm{shift},\, \tilde{t}}, \end{aligned}$$

where $\Delta V^{2\ell ,\,\tilde{t}}$ denotes the contribution to the MSSM scalar potential from two-loop diagrams involving the strong gauge interactions of the stop squarks, given e.g. in Eq. (28) of Ref. [66]. The derivatives of $\Delta V^{2\ell ,\,\tilde{t}}$ are again obtained from Eq. (25) after expressing the stop masses and mixing angle as functions of the field-dependent top mass. As in the case of the two-loop matching condition for the quartic Higgs coupling discussed in Sect. 2.1, in the derivatives of the two-loop scalar potential we find terms proportional to $\ln (m_t^2/Q^2)$, which would diverge in the limit of vanishing top mass. Those terms, however, cancel against analogous terms in $\delta c_6^{\scriptscriptstyle \mathrm{EFT}}$, which represents the one-loop contribution of the dimension-six operators to the Wilson coefficient of $|H|^6$ as computed in the EFT. In particular, the contribution relevant at $\mathcal{O}(g_t^6g_3^2)$ arises from a box diagram with a top-quark loop, three regular Yukawa vertices and one dimension-six vertex. We find

29 $$\begin{aligned} \delta c_6^{\scriptscriptstyle \mathrm{EFT}}= -\frac{N_c}{(4\pi )^2}\,g_t^3\,c_t^{1\ell }(Q) \left( 4\,\ln \frac{m_t^2}{Q^2}+\frac{32}{3}\right) , \end{aligned}$$

where for $c_t^{1\ell }(Q)$ we use the $\mathcal{O}(g_t^3\,g_3^2)$ contribution to the matching condition given in Eq. (21). Finally, the third term on the right-hand side of Eq. (28) arises from the fact that, in analogy with our two-loop calculation of the quartic Higgs coupling, we choose to express the one-loop stop contribution to $c_6^{1\ell }$ in terms of the $\overline{\mathrm{MS}}$-renormalized top Yukawa coupling of the EFT, i.e. $g_t$, as opposed to the ${ \overline{\mathrm{DR}}}$-renormalized coupling of the MSSM, i.e. the $\hat{g}_t$ entering Eqs. (25)–(27).^10^ The resulting $\mathcal{O}(g_t^6\,g_3^2)$ shift in $c_6^{2\ell }$ reads

30 $$\begin{aligned} \delta c_6^{\mathrm{shift},\,\tilde{t}} = 6\,\Delta g_t^s\,c_6^{1\ell }(Q), \end{aligned}$$

where $\Delta g_t^s$ is given in Eq. (20) and $c_6^{1\ell }(Q)$ is given in Eq. (26).

The analytic formula for $c_6^{2\ell }(Q)$ for generic stop and gluino masses is too lengthy to be printed, and we make it available on request in electronic form. For the simplified choice $m_{Q_3}=m_{U_3}=m_{\tilde{g}}=M_S$, we obtain

31 $$\begin{aligned} c_6^{2\ell }(Q)= & {} -\frac{\hat{g}_t^6\,g_3^2}{(4\pi )^4}\,\frac{C_FN_c}{M_S^2} \left[ \frac{2}{3}-\frac{4\,X_t}{M_S}+ \frac{X_t^2}{M_S^2} +\frac{14\,X_t^3}{3\,M_S^3}\right. \nonumber \\&+\,\left. \frac{X_t^4}{6\,M_S^4} -\frac{13\,X_t^5}{10\,M_S^5}-\frac{19\,X_t^6}{180\,M_S^6} +\frac{X_t^7}{10\,M_S^7}\right. \nonumber \\&\left. +\,\left( \frac{8}{3}+\frac{2\,X_t}{M_S}- \frac{4\,X_t^2}{M_S^2} -\frac{2\,X_t^3}{M_S^3}\right. \right. \nonumber \\&\left. \left. +\,\frac{X_t^4}{M_S^4} +\frac{2\,X_t^5}{5\,M_S^5}-\frac{X_t^6}{30\,M_S^6}\right) \ln \frac{M_S^2}{Q^2}\,\right] . \end{aligned}$$

We also remark that $c_6^{2\ell }(Q)$ contains terms enhanced by powers of the ratios between the gluino mass and the stop masses. In particular, in the simplified scenario where $m_{Q_3}=m_{U_3}=M_S$, $\,X_t= \pm \sqrt{6}\,M_S$ and $m_{\tilde{g}}\gg M_S$ we find

32 $$\begin{aligned} c_6^{2\ell }(Q)= & {} -\frac{\hat{g}_t^6\,g_3^2}{(4\pi )^4}\,\frac{4\,C_FN_c}{15\,M_S^2} \left[ 1 \,-\, 18\,\ln \frac{M_S^2}{Q^2} \,+\, 13\,\ln \frac{m_{\tilde{g}}^2}{M_S^2} \,\right. \nonumber \\&-\left. \, \frac{m_{\tilde{g}}}{M_S}\left( \pm \, 9 \,\sqrt{6} \, +\, 31\,\frac{m_{\tilde{g}}}{M_S}\right) \!\left( 1-\ln \frac{m_{\tilde{g}}^2}{Q^2} \right) \right. \nonumber \\&\left. +\,\mathcal{O}\left( \frac{M_S}{m_{\tilde{g}}}\right) \,\right] , \end{aligned}$$

where the sign of the first term within round brackets corresponds to the sign of $X_t$. The presence of power-enhanced terms in the heavy-gluino limit is a well-known consequence of the ${ \overline{\mathrm{DR}}}$ renormalization of the parameters in the stop sector, as discussed in Ref. [22] for the fixed-order calculation of the MSSM Higgs masses and in Ref. [67] for the EFT calculation. Those terms would be removed from the two-loop part of $c_6$ if we interpreted the soft SUSY-breaking stop masses $m_{Q_3}$ and $m_{U_3}$ and the stop mixing $X_t$ entering the one-loop part as “on-shell”-renormalized parameters.

**Comparison with the fixed-order calculation of** $\mathbf {m_h^2}$: We now discuss how the inclusion in the EFT Lagrangian of the dimension-six operators shown in Eq. (16) allows us to reproduce the $\mathcal{O}(m_t^2/M_S^2)$ terms in the $\mathcal{O}(\alpha _t)$ and $\mathcal{O}(\alpha _t\alpha _s)$ corrections to $m_h^2\,$. Expanding the neutral component of the Higgs doublet as $H^0 = v + (h\,+\,i\,G)/\sqrt{2}\,$, and exploiting the minimum condition of the scalar potential to remove the mass parameter $m_{{\scriptscriptstyle H}}^2$, we can write the Higgs-boson mass as

33 $$\begin{aligned} m_h^2 = 2\,\lambda \,v^2 + 12\, c_6\,v^4 + \Delta m_h^2, \end{aligned}$$

where $\Delta m_h^2$ contains the radiative corrections to the tree-level prediction for the Higgs mass, as computed in the EFT. To avoid the occurrence of large logarithms in these corrections, the couplings $\lambda$ and $c_6$ in Eq. (33) should be computed at a renormalization scale $Q_{\mathrm{{\scriptscriptstyle EW}}}$ of the order of the masses of the particles running in the loops. Focusing on the one- and two-loop terms that account for the desired $\mathcal{O}(\alpha _t)$ and $\mathcal{O}(\alpha _t\alpha _s)$ corrections, we find

34 $$\begin{aligned} \Delta m_h^2= & {} \frac{N_c}{(4\pi )^2}\,\left[ -4\,g_t^4\,v^2\,\ln \frac{g_t^2\,v^2}{Q_{\mathrm{{\scriptscriptstyle EW}}}^2}\right. \nonumber \\&+\,\left. 8\,g_t\,c_t\,v^2\,m_t^2\,\left( 1-6\,\ln \frac{m_t^2}{Q_{\mathrm{{\scriptscriptstyle EW}}}^2}\right) \, \right] \nonumber \\&+\, \frac{C_F N_c}{(4\pi )^4}\,8\,g_t^2\,g_3^2\,m_t^2 \left( 3\,\ln ^2\frac{m_t^2}{Q_{\mathrm{{\scriptscriptstyle EW}}}^2}~+~\ln \frac{m_t^2}{Q_{\mathrm{{\scriptscriptstyle EW}}}^2}\right) ,\nonumber \\ \end{aligned}$$

where the first two lines are the contribution of one-loop diagrams involving top quarks, the third line is the contribution of two-loop diagrams involving top quarks and gluons computed in the $\overline{\mathrm{MS}}$ scheme (the latter was given, e.g., in Ref. [96]), and we can typically take $Q_{\mathrm{{\scriptscriptstyle EW}}}\approx m_t$. Since for the purpose of this calculation the coefficient $c_t$ is first generated at one loop, in the first two lines of Eq. (34) we have exploited the relation $m_t= g_t\,v + c_t\,v^3$ and retained^11^ only terms linear in $c_t$ (note that in those terms, as well as in those of the third line, the difference between $m_t^2$ and $g_t^2\,v^2$ amounts to a higher-order effect). Collecting all the terms in Eq. (33) that involve the coefficients of dimension-six operators, we thus find for the one- and two-loop $\mathcal{O}(m_t^2/M_S^2)$ terms

35 $$\begin{aligned} \left( m_h^2\right) _{\scriptscriptstyle \mathrm{dim6}}^{\mathcal{O}(\alpha _t)}= & {} 12\,v^4 \,c_6^{1\ell }(Q),\end{aligned}$$

36 $$\begin{aligned} \left( m_h^2\right) _{\scriptscriptstyle \mathrm{dim6}}^{\mathcal{O}(\alpha _t\alpha _s)}= & {} 12\,v^4 \left[ c_6^{2\ell }(Q) ~+ \left. \frac{\mathrm{d} c_6}{\mathrm{d} \ln Q^2}\right| _{g_t^3c_t} \! \ln \frac{Q_{\mathrm{{\scriptscriptstyle EW}}}^2}{Q^2}\right] \nonumber \\&+\, \frac{N_c}{(4\pi )^2}\,8\,g_t\,c_t\,v^2\,m_t^2\, \left( 1-6\,\ln \frac{m_t^2}{Q_{\mathrm{{\scriptscriptstyle EW}}}^2}\right) ,\nonumber \\ \end{aligned}$$

where the one-loop beta function of $c_6$ entering the squared brackets in Eq. (36) accounts, at the two-loop level, for the fact that in Eq. (33) the coefficient $c_6$ should be computed at the low scale $Q_{\mathrm{{\scriptscriptstyle EW}}}$. Isolating the relevant terms in the RGEs for the dimension-six operators given in Refs. [97–100], we have

37 $$\begin{aligned} \frac{\mathrm{d} c_6}{\mathrm{d} \ln Q^2}~\supset ~ -4\,N_c\,\frac{g_t^3\,c_t}{(4\pi )^2},\quad \frac{\mathrm{d} c_t}{\mathrm{d} \ln Q^2}~\supset ~ -3\,C_F\,\frac{g_3^2\,c_t}{(4\pi )^2}. \end{aligned}$$

However, for the coefficient $c_t$ entering Eq. (36) we can use directly the value obtained at the matching scale, see Eq. (21), because its scale dependence amounts to a three-loop effect in $m_h^2$. We thus obtain

38 $$\begin{aligned} \left( m_h^2\right) _{\scriptscriptstyle \mathrm{dim6}}^{\mathcal{O}(\alpha _t\alpha _s)}= & {} 12\, v^4\, c_6^{2\ell }(Q)\nonumber \\&+ \frac{N_c}{(4\pi )^2}\,8\,g_t\,c_t^{1\ell }(Q)\,v^2\,m_t^2\, \left( 1-6\,\ln \frac{m_t^2}{Q^2}\right) .\nonumber \\ \end{aligned}$$

Expanding in powers of $m_t^2$ the analytic results of Ref. [22] for the $\mathcal{O}(\alpha _t)$ and $\mathcal{O}(\alpha _t\alpha _s)$ corrections to $m_h^2$ in the MSSM, we checked that Eqs. (35) and (38) do indeed reproduce the one- and two-loop $\mathcal{O}(m_t^2/M_S^2)$ terms of those corrections, respectively. To this purpose, it is necessary to take into account that in Ref. [22] the top mass and Yukawa coupling entering the one-loop part of the corrections to $m_h^2$ are assumed to be MSSM parameters renormalized in the ${ \overline{\mathrm{DR}}}$ scheme, whereas, as discussed earlier, we choose to express $c_6^{1\ell }(Q)$ in terms of the EFT coupling $g_t$ renormalized in the $\overline{\mathrm{MS}}$ scheme. To perform the comparison with the fixed-order calculation of $m_h^2$ we must therefore omit the term $\delta c_6^{\mathrm{shift},\,\tilde{t}}$ in our formula for $c_6^{2\ell }(Q)$; see Eqs. (28) and (30).

### Impact of dimension-six operators on the Higgs-mass prediction

In this section we illustrate the numerical impact of the dimension-six operators of Eq. (16) on the EFT prediction for the Higgs mass. We modified the code HSSUSY [72], implementing the matching conditions for $c_6$ and $c_t$ at the SUSY scale, their evolution down to the EW scale through the RGEs of Eq. (37),^12^ and their effects at the EW scale, both on the calculation of $m_h^2$ – see Eqs. (33) and (34) – and on the determination of the top Yukawa coupling. In particular, the latter becomes

39 $$\begin{aligned} g_t(Q_{\mathrm{{\scriptscriptstyle EW}}}) = \frac{\overline{m}_t}{v}- c_t\,v^2, \end{aligned}$$

where $\overline{m}_t$ denotes the $\overline{\mathrm{MS}}$-renormalized top mass, extracted at the scale $Q_{\mathrm{{\scriptscriptstyle EW}}}$ from $M_t^{\scriptscriptstyle \mathrm{pole}}$ with SM formulas, and we neglect the effects of dimension-six operators that do not contribute at $\mathcal{O}(g_3^2)$. We note that the $c_t$-induced shift on the matching condition for the top Yukawa coupling, Eq. (39) above, affects all corrections controlled by $g_t$ to the quartic Higgs coupling – namely, the threshold corrections at the SUSY scale and the renormalization-group evolution down to the EW scale – as well as the top-quark contributions to $\Delta m_h^2$ given in Eq. (34). This results in an “indirect” contribution of a dimension-six operator to the EFT prediction for $m_h^2$, which combines with the “direct” contributions controlled by $c_6$ and $c_t$ in Eqs. (33) and (34).

**Fig. 3:**
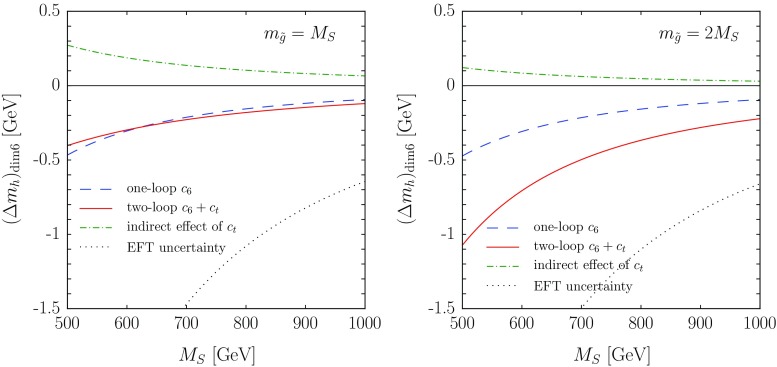
Effects of dimension-six operators on the EFT prediction for the Higgs mass, as a function of a common stop mass scale $M_S$, for $X_t = \sqrt{6}\,M_S$. In the *left plot* we take $m_{\tilde{g}}=M_S$, whereas in the *right plot* we take $m_{\tilde{g}}=2\,M_S$. The meaning of the *different curves* and the values of the remaining MSSM parameters are described in the text

**Fig. 4:**
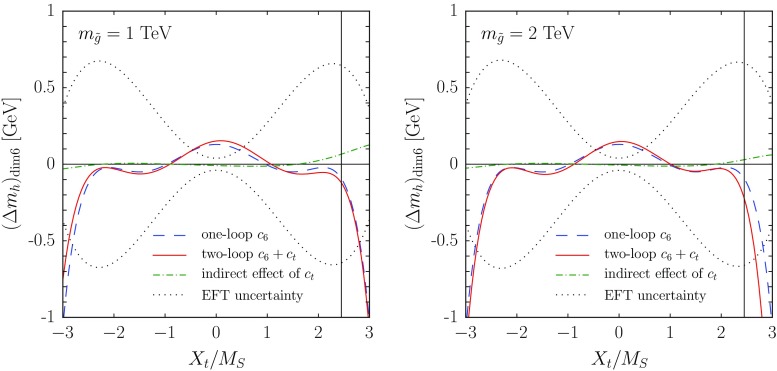
Effects of dimension-six operators on the EFT prediction for the Higgs mass, as a function of the ratio $X_t/M_S$, for a common stop mass scale $M_S= 1~\mathrm{TeV}$. In the *left plot* we take $m_{\tilde{g}}=1~\mathrm{TeV}$, whereas in the *right plot* we take $m_{\tilde{g}}=2~\mathrm{TeV}$. The *thin vertical lines* in the two plots mark the condition $X_t/M_S= \sqrt{6}\,$. The meaning of the *different curves* and the values of the remaining MSSM parameters are the same as in Fig. 3, as described in the text

In Fig. 3 we show the deviation induced in the EFT prediction for the Higgs mass by the presence of the dimension-six operators of Eq. (16). The SM parameters used as input for HSSUSY are the same as those listed at the beginning of Sect. 2.2. We consider a simplified MSSM scenario with $\tan \beta =20$ and all soft SUSY-breaking masses of sfermions and EW gauginos, as well as the heavy Higgs-doublet mass $m_{\scriptscriptstyle A}$ and the higgsino mass $\mu$, set equal to a common SUSY scale $M_S$; the trilinear Higgs-stop coupling $A_t$ is fixed by the maximal mixing condition $A_t-\mu \,\cot \beta = \sqrt{6}\,M_S\,$, and $A_b=A_{\tau }=A_t$; finally, the gluino mass is set to $m_{\tilde{g}}= M_S$ in the left plot and to $m_{\tilde{g}}= 2\,M_S$ in the right plot. We vary the common SUSY scale between $M_S= 500$ GeV and $M_S= 1$ TeV, and interpret the soft SUSY-breaking stop masses and $A_t$ as ${ \overline{\mathrm{DR}}}$-renormalized parameters at the matching scale $Q=M_S$. We remark that, in the considered range of $M_S$, the prediction of HSSUSY for $m_h$ (before the introduction of the dimension-six operators) varies between 120.2 and 123 GeV in the left plot and between 118.7 and 121.9 GeV in the right plot, always several GeV below the value measured at the LHC. Therefore, rather than depicting fully realistic scenarios, the figure is meant to illustrate the relative importance of the different effects induced by dimension-six operators, and how those effects get suppressed by an increase in the SUSY scale.

The dashed blue lines in the plots of Fig. 3 represent the inclusion of the sole operator $|H|^6$, with the coefficient $c_6$ computed at one loop and “frozen” at the matching scale $Q=M_S$. This accounts for the $\mathcal{O}(m_t^2/M_S^2)$ part of the $\mathcal{O}(\alpha _t)$ corrections to the Higgs mass, as given in Eq. (35). We see that, in these scenarios, the corresponding shift in $m_h$ is negative and rather modest, decreasing from about 470 MeV for $M_S=500$ GeV to about 90 MeV for $M_S=1$ TeV.

The solid red lines in the plots of Fig. 3 represent instead the inclusion of both of the operators of Eq. (16), with coefficients $c_6$ and $c_t$ computed at two loops and one loop, respectively, and evolved between the scales $M_S$ and $Q_{\mathrm{{\scriptscriptstyle EW}}}$ with the RGEs of Eq. (37). This accounts also for the $\mathcal{O}(m_t^2/M_S^2)$ part of the $\mathcal{O}(\alpha _t\alpha _s)$ corrections to the Higgs mass, as given in Eq. (38). In the scenario shown in the left plot, corresponding to $m_{\tilde{g}}= M_S$, the two-loop $\mathcal{O}(m_t^2/M_S^2)$ corrections appear to be rather small, never reaching even $\pm 100$ MeV in the considered range of $M_S$. However, we must take into account that the difference between the dashed blue and solid red lines results from the combination of several effects, namely: (i) the $c_t$-induced shift in the value of $g_t$ used in the whole calculation, see Eq. (39), whose “indirect” effects on $m_h$ we show for illustration as the dot-dashed green lines; (ii) the inclusion of the two-loop part of the matching condition for $c_6$ at the SUSY scale; (iii) the evolution of $c_6$ (and of $c_t$) between the SUSY scale and the EW scale; (iv) the terms controlled by $c_t$ in the radiative corrections to the Higgs mass at the EW scale; see Eq. (34). In the scenario of the left plot, the first three of these effects shift $m_h$ by several hundred MeV each for $M_S=500$ GeV, but they undergo significant cancellations, whereas the fourth effect is considerably less important. On the other hand, in the scenario shown in the right plot, corresponding to $m_{\tilde{g}}= 2\,M_S$, the “indirect” effects of the shift in $g_t$ are reduced due to a smaller value of $c_t$, and the two-loop contribution to the matching of $c_6$ at the SUSY scale doubles in size and changes sign, with the result that the combined effects of the two-loop $\mathcal{O}(m_t^2/M_S^2)$ corrections are much more significant than in the left plot, further decreasing the prediction for $m_h$ by about 600 MeV for $M_S=500$ GeV and about 130 MeV for $M_S=1$ TeV.

To assess the relevance of the $\mathcal{O}(m_t^2/M_S^2)$ logarithmic effects beyond two loops, we removed from the EFT prediction for the Higgs mass the higher-order terms that are picked up by solving numerically the RGEs of the Wilson coefficients in Eq. (37). In practice, we compared our results for $m_h$ with those obtained by “freezing” $c_t$ at the SUSY scale and truncating the evolution of $c_6$ to the first order in the perturbative expansion – see the terms within square brackets in Eq. (36). We found that these higher-order logarithmic effects are very small in the considered scenarios: even in the one with $m_{\tilde{g}}=M_S$, characterized by a larger value of $c_t$ and hence a stronger scale dependence of both $c_t$ and $c_6$, the resulting shift in $m_h$ reaches a maximum of about 20 MeV for $M_S\approx 600$ GeV, then decreases for larger $M_S$ as the suppression by a factor $m_t^2/M_S^2$ begins to prevail over the logarithmic enhancement.

Finally, the dotted black lines in the plots of Fig. 3 represent a naive estimate of the overall size of the $\mathcal{O}(v^2/M_S^2)$ corrections to the Higgs mass, corresponding to the “EFT uncertainty” implemented in the code $\mathtt{SusyHD}$. Following Ref. [67], we obtain that estimate by multiplying the contribution to $\Delta \lambda ^{1\ell }$ from each SUSY particle with mass $M_i$ by a factor^13^ $(1 \pm 2\, v^2/M_i^2)$. It appears that, even in the scenario of the right plot where the computed $\mathcal{O}(m_t^2/M_S^2)$ corrections to the Higgs mass are more significant, the $\mathtt{SusyHD}$ estimate of those effects is larger by about a factor of three. Therefore, even if the one- and two-loop $\mathcal{O}(v^2/M_S^2)$ corrections to $m_h^2$ that are not included in our analysis – such as, e.g., the two-loop corrections proportional to $g_t^4\,m_t^4/M_S^2$ – were as large as the ones that we did compute and had the same sign, the estimate of the “EFT uncertainty” implemented in $\mathtt{SusyHD}$ would turn out to be sufficiently conservative in the considered scenarios.

It is legitimate to wonder whether the relatively small size of the $\mathcal{O}(m_t^2/M_S^2)$ corrections found in the scenarios of Fig. 3 is just an accident, perhaps related to the choice $X_t = \sqrt{6}\,M_S$ made to ensure a near-maximal prediction for the Higgs mass. To answer this question, in Fig. 4 we show again the deviation induced in the EFT prediction for the Higgs mass by the presence of the dimension-six operators of Eq. (16), this time as a function of the ratio $X_t/M_S$. We set $M_S=1$ TeV, and take all of the remaining MSSM parameters as in the two scenarios of Fig. 3. In particular, we take $m_{\tilde{g}}=1$ TeV in the left plot and $m_{\tilde{g}}=2$ TeV in the right plot. The thin vertical lines in the two plots of Fig. 4 mark the condition $X_t/M_S= \sqrt{6}$, i.e. they map the right edge of the corresponding plots in Fig. 3. The meaning of all other lines is the same as in Fig. 3.

Figure 4 shows that, for a given value of $M_S$, the impact of the $\mathcal{O}(m_t^2/M_S^2)$ corrections to the Higgs mass can indeed be larger than the one found when $X_t/M_S= \sqrt{6}\,$. This happens in particular for $X_t \approx 0$, or for values of $|X_t/M_S|$ larger than $\sqrt{6}$. The figure also shows that for $X_t \approx 0$ the $\mathtt{SusyHD}$ estimate of the “EFT uncertainty” falls short of the computed $\mathcal{O}(m_t^2/M_S^2)$ corrections to the Higgs mass. Indeed, the main contribution to the $\mathtt{SusyHD}$ estimate is the one from stops, which – being proportional to the corresponding contribution to $\Delta \lambda ^{1\ell }$, see Eq. (2) – is maximized for $|X_t/M_S| = \sqrt{6}\,$ and vanishes for $X_t=0$ (the small non-zero value of the “EFT uncertainty” visible in the plots at $X_t=0$ is due to the contributions of EW gauginos and higgsinos). In contrast, Eq. (27) shows that the one-loop stop contribution to $c_6$ does not vanish for $X_t=0$, yielding a shift in $m_h$ of about 130 MeV. However, we must recall that moving away from the “maximal mixing” condition on $X_t$ results in a significant decrease in the EFT prediction for the Higgs mass, e.g. for $X_t = 0$ we would find $m_h\approx 110.8$ GeV. In order to recover a prediction for $m_h$ within a few GeV from the observed value, we would need to raise the SUSY scale $M_S$ to several TeV, strongly suppressing all effects of dimension-six operators. Therefore, the $\mathtt{SusyHD}$ estimate of the “EFT uncertainty” happens to be at its most conservative precisely in the region of the MSSM parameter space where the $\mathcal{O}(m_t^2/M_S^2)$ effects discussed in this section have a chance to be numerically relevant.

## Conclusions

If the MSSM is realized in nature, both the measured value of the Higgs mass and the (so far) negative results of the searches for superparticles at the LHC suggest some degree of separation between the SUSY scale $M_S$ and the EW scale. In this scenario the MSSM prediction for the Higgs mass is subject to potentially large logarithmic corrections, which can be resummed to all orders in an EFT approach. Over the past few years this has stimulated a considerable amount of activity, aimed, on one hand, at refining the EFT calculation of the MSSM Higgs mass [65–67], and, on the other hand, at combining it with the fixed-order calculations implemented in public codes for the determination of the MSSM mass spectrum [64, 73, 75, 76]. Here we contributed to these efforts by providing a complete determination of the two-loop threshold corrections to the quartic Higgs coupling in the limit of vanishing EW gauge (and first-two-generation Yukawa) couplings, for generic values of all the relevant SUSY-breaking parameters. We also studied a class of one- and two-loop corrections to the Higgs mass suppressed by $m_t^2/M_S^2\,$, extending the SM Lagrangian with appropriate dimension-six operators. All of our results are available upon request in electronic form, and they were also implemented in modified versions of the codes SusyHD [69] and HSSUSY [72].

The numerical impact of the various corrections computed in this paper turns out to be small, typically below one GeV in regions of the MSSM parameter space where the prediction for the Higgs mass is within a few GeV from the observed value. We stress that this is in fact a desirable feature of the EFT calculation of the Higgs mass: while the logarithmically enhanced corrections are accounted for by the evolution of the parameters between the matching scale and the EW scale, and high-precision calculations at the EW scale can be borrowed from the SM, the small impact of the two-loop corrections computed at the matching scale suggests that the “SUSY uncertainty” associated to uncomputed higher-order terms should be well under control. In principle, the advantages of an EFT approach are less clear-cut when there is only a moderate separation between the SUSY scale and the EW scale, so that the omission of $\mathcal{O}(v^2/M_S^2)$ effects in the calculation of the Higgs mass is not warranted. However, our study of the dimension-six operators suggests that the naive estimate of the theoretical uncertainty associated to missing $\mathcal{O}(v^2/M_S^2)$ effects (or “EFT uncertainty”) implemented in the code SusyHD is indeed sufficiently conservative in the relevant regions of the MSSM parameter space. The EFT approach also becomes more complicated when some of the new particles are much lighter than the rest. For example, while our results for the two-loop corrections to the quartic Higgs coupling can be directly applied to the standard split-SUSY scenario by taking the limit of vanishing gluino and higgsino masses, scenarios in which both Higgs doublets are light require a dedicated calculation, in which the effective theory valid below the SUSY scale is a THDM (see, e.g., Ref. [71]).

Finally, we recall that the accuracy of the measurement of the Higgs mass at the LHC has already reached the level of a few hundred MeV – i.e., comparable to the effects of the corrections discussed in this paper – and will improve further when more data become available. If SUSY shows up at last, the mass and the couplings of the SM-like Higgs boson will serve as precision observables to constrain MSSM parameters that might not be directly accessible by experiment, especially in scenarios where some of the superparticle masses are in the multi-TeV range. To this purpose, the accuracy of the theoretical predictions will have to match the experimental one, making a full inclusion of two-loop effects in the Higgs-mass calculation unavoidable. Our results should be regarded as necessary steps in that direction.

## Appendix

We present here the one-loop scalar contributions to the matching condition for the quartic Higgs coupling, including all terms controlled by third-family Yukawa couplings:

A1 $$\begin{aligned} (4\pi )^2\,\Delta \lambda ^{1\ell ,\,\phi }= & {} N_c\, \hat{g}_t^2 \left[ \hat{g}_t^2 \,+\, \frac{1}{2} \left( g_2^2-\frac{g_1^2}{5}\right) \cos 2 \beta \right] \ln \frac{m_{Q_3}^2}{Q^2}\nonumber \\&+\,N_c\, \hat{g}_t^2 \left[ \hat{g}_t^2 + \frac{2}{5}\, g_1^2\, \cos 2 \beta \right] \ln \frac{m_{U_3}^2}{Q^2} \nonumber \\&+\, N_c\, \hat{g}_b^2 \left[ \hat{g}_b^2 \,-\, \frac{1}{2} \left( g_2^2+\frac{g_1^2}{5}\right) \cos 2 \beta \right] \ln \frac{m_{Q_3}^2}{Q^2}\nonumber \\&+\,N_c\, \hat{g}_b^2 \left[ \hat{g}_b^2 - \frac{g_1^2}{5}\,\, \cos 2 \beta \right] \ln \frac{m_{D_3}^2}{Q^2} \nonumber \\&+\, \hat{g}_\tau ^2 \left[ \hat{g}_\tau ^2 \,-\, \frac{1}{2} \left( g_2^2 - \frac{3}{5}\,g_1^2 \right) \cos 2 \beta \right] \ln \frac{m_{L_3}^2}{Q^2}\nonumber \\&+\, \hat{g}_\tau ^2 \left[ \hat{g}_\tau ^2 - \frac{3}{5}\, g_1^2\,\, \cos 2 \beta \right] \ln \frac{m_{E_3}^2}{Q^2} \nonumber \\&+\,\frac{ \cos ^2 2 \beta }{300}\, \sum _{i=1}^3\, \bigg [\,N_c\left( g_1^4+25\, g_2^4\right) \, \ln \frac{m_{Q_i}^2}{Q^2} \,\nonumber \\&+\,8\,N_c\, g_1^4 \,\ln \frac{m_{U_i}^2}{Q^2} \,+\,2\,N_c\, g_1^4 \,\ln \frac{m_{D_i}^2}{Q^2} \nonumber \\&+\, \left( 9 \,g_1^4+25\, g_2^4\right) \, \ln \frac{m_{L_i}^2}{Q^2} \,\nonumber \\&+\,18 \,g_1^4 \,\ln \frac{m_{E_i}^2}{Q^2} \bigg ] \nonumber \\&+\,\frac{1}{4800}\, \bigg [261\, g_1^4+630\, g_1^2 g_2^2 +1325\, g_2^4\nonumber \\&-\, 4 \,\cos 4 \beta \left( 9\, g_1^4+90\, g_1^2 g_2^2+175\, g_2^4\right) \nonumber \\&-\,9\, \cos 8\beta \left( 3\, g_1^2+5\, g_2^2\right) ^2 \bigg ] \ln \frac{m_{\scriptscriptstyle A}^2}{Q^2}\nonumber \\&-\,\frac{3}{16} \left( \frac{3}{5} g_1^2 + g_2^2\right) ^2 \sin ^2 4 \beta \nonumber \\&+\, \sum _{f=t,b,\tau }~\hat{g}_f^2 \, N^f_c\, \widetilde{X}_f \left\{ 2\,\hat{g}_f^2 \, \Big [\widetilde{F}_1\left( x_f\right) \right. \nonumber \\&-\frac{\widetilde{X}_f}{12} \,\widetilde{F}_2\left( x_f\right) \Big ] \nonumber \\&+\, \frac{\cos 2\beta }{4} \, \left[ \,\frac{9}{10} \, g_1^2 \,Q_f\, \widetilde{F}_3 \left( x_f\right) \right. \nonumber \\&\left. +\, \left( 2\, g_2^2 \,T^3_{f_{\scriptscriptstyle L}} + \frac{3}{5}\,g_1^2\,(2\,T^3_{f_{\scriptscriptstyle L}}\right. \right. \nonumber \\&\left. \left. -\,\frac{3}{2} \,Q_f )\right) \widetilde{F}_4 \left( x_f\right) \right] \nonumber \\&\left. -\,\frac{\cos ^2 2\beta }{12} \, \left( \frac{3}{5} g_1^2 +g_2^2 \right) \widetilde{F}_5\left( x_f \right) ~\right\} , \end{aligned}$$

where the compact notation used in the sum over the sfermion species $f=t,b,\tau$ is described after Eq. (2), and all loop functions $\widetilde{F}_i$ are defined in Appendix A of Ref. [66]. In addition, $Q_f$ is the electric charge and $T^3_{f_{\scriptscriptstyle L}}$ is the third component of the weak isospin of the “left” sfermion of each species. We recall that Eq. (A1) assumes that the tree-level part of the matching condition for $\lambda \,$, see Eq. (1), be expressed in terms of the EW gauge couplings of the SM and of an angle $\beta$ defined as in Sect. 2.2 of Ref. [66]. We also remark that the third-family Yukawa couplings $\hat{g}_f$ entering Eq. (A1) are the MSSM ones. As discussed in Sect. 2.1, our choice of using instead the top and tau Yukawa couplings of the SM (denoted as $g_t$ and $g_\tau$) in the one-loop part of the threshold correction to the quartic Higgs coupling induces shifts in the two-loop part of the correction; see Eq. (11). Finally, we note that Eq. (A1) differs from Eq. (11) of Ref. [67] by the presence of the terms in the third to sixth lines.

## Footnotes

1 We focus here on the MSSM with real parameters. Significant efforts have also been devoted to the Higgs-mass calculation in the presence of CP-violating phases, as well as in non-minimal SUSY extensions of the SM.

2 See, however, Ref. [60] for the effect of dimension-six operators in a scenario with only one light stop.

3 Refs. [64, 65] also obtained analytic results for the coefficients of logarithmic terms in $\Delta m_h^2$ beyond three loops.

4 Beyond tree level, we must distinguish these couplings from the proper Yukawa couplings of the SM, denoted as $g_f\,$, and specify a renormalization prescription for the angle $\beta$.

5 We compared our result for the top and bottom Yukawa contribution to $\Delta V^{2\ell ,\,\mathrm{heavy}}$ with the one obtained by imposing the gaugeless limit and removing the SM-like contribution in Eq. (D.6) of Ref. [21]. We find agreement except for the overall sign of the next-to-last line of that equation.

6 Note that in this paper we normalize the Higgs vev as $v = \langle H^0\rangle$, with $v\approx 174$ GeV.

7 For those operators several definitions are possible. For example, in Ref. [90] one chose $(H^{\dagger }H)\Box (H^{\dagger }H)$ and $(H^{\dagger }D_\mu \,H)^*(H^{\dagger }D_\mu \, H)$.

8 Focusing on the CP-even operators, those are $f^{abc}\,G_{\mu \nu }^a\,G_{\nu \rho }^b\,\,G_{\rho \mu }^c\,$, $|H|^2\,G_{\mu \nu }^a\,G_{\mu \nu }^a$  and  $\overline{t_{\scriptscriptstyle R}} \, \sigma ^{\mu \nu } \,T^a\,H^{\scriptscriptstyle T}\epsilon \, q_{\scriptscriptstyle L}\,G_{\mu \nu }^a\,$.

9 In Ref. [92] there is a misprint in the last line of Eq. (D.4): the logarithmic term should come with a minus sign.

10 On the other hand, in the two-loop corrections the distinction between $g_t$ and $\hat{g}_t$ amounts to a higher-order effect.

11 Before the expansion in $c_t$ the one-loop contribution to $\Delta m_h^2$ involving top quarks reads, in our EFT,

$$\begin{aligned} (\Delta m_h^2)^{1\ell ,\,t}= & {} \frac{N_c}{(4\pi )^2}\,\left[ \, 2\,m_t^2\,\left( g_t+3\,c_t\,v^2\right) ^2 \left( 1-3\,\ln \frac{m_t^2}{Q_{\mathrm{{\scriptscriptstyle EW}}}^2}\right) \,\right. \\&-\,\left. \, 2\,\frac{m_t^3}{v}\,\left( g_t-3\,c_t\,v^2\right) \left( 1-\ln \frac{m_t^2}{Q_{\mathrm{{\scriptscriptstyle EW}}}^2} \right) \,\right] . \end{aligned}$$

12 Note that we neglect additional terms in those RGEs, as well as the contributions of the dimension-six operators to the RGEs of the SM couplings [98], because they do not contribute to the $\mathcal{O}(\alpha _t)$ and $\mathcal{O}(\alpha _t\alpha _s)$ corrections to $m_h^2$.

13 To be conservative, we adjust the signs in the rescaling factors for scalars and EW-inos so that the resulting shifts in $\Delta \lambda ^{1\ell }$ add up. The upper edge of the uncertainty band, not shown in the plots, can be obtained by reversing all signs.
